# Interpretable Machine Learning for Predicting Metabolic Syndrome–Kidney Stone Disease Comorbidity: The Role of Dietary Micronutrients

**DOI:** 10.1002/fsn3.72019

**Published:** 2026-06-10

**Authors:** Guanwei Wu, Haibo Qin, Junfeng Yao, Yingqing Liu, Jie Zheng, Jiawei Wang, Zongyao Hao, Lingsong Tao

**Affiliations:** ^1^ Department of Urology Affiliated Wuhu Hospital of East China Normal University Wuhu Anhui China; ^2^ Department of Urology The First Affiliated Hospital of Anhui Medical University Hefei Anhui China

**Keywords:** dietary micronutrients, kidney stone disease, machine learning, metabolic syndrome, NHANES

## Abstract

This study aimed to develop interpretable machine‐learning models to predict the risk of metabolic syndrome–kidney stone disease (MetS–KSD) comorbidity based on dietary micronutrient intake. Using data from the National Health and Nutrition Examination Survey (NHANES) from 2007 to 2018, 54 candidate features, including dietary variables and demographic covariates, were incorporated into the analysis. Six mainstream machine‐learning models, including Random Forest, XGBoost, LightGBM, k‐nearest neighbors, support vector machine, and Naïve Bayes, were evaluated using a comprehensive multi‐metric framework incorporating the area under the receiver operating characteristic curve (AUC‐ROC), area under the precision–recall curve (AUC‐PR), accuracy, F‐beta score, sensitivity, and specificity. SHapley Additive exPlanations (SHAP) and Local Interpretable Model‐agnostic Explanations (LIME) were applied to enhance model interpretability. A total of 4936 participants were included, representing approximately 4,597,435 U.S. adults with MetS–KSD comorbidity after application of sampling weights. After variance inflation factor analysis and Boruta feature selection, 33 features were retained, including 25 dietary micronutrients and 8 demographic variables. When demographic and dietary variables were jointly modeled, Random Forest demonstrated the best performance (AUC‐ROC = 0.958; AUC‐PR = 0.961). When models were constructed using dietary micronutrients alone, XGBoost achieved optimal performance (AUC‐ROC = 0.956; AUC‐PR = 0.960). SHAP and LIME analyses identified lycopene, added vitamin B12, magnesium, dietary fiber, theobromine, and vitamin K as key contributing features, with importance rankings varying according to demographic context. Supplementary sensitivity analyses further supported model robustness and the effectiveness of SMOTE‐based imbalance correction. These findings suggest that interpretable machine learning may provide a useful framework for nutritional risk stratification in MetS–KSD comorbidity, although external validation in independent populations is still required.

## Graphical abstract

This study used NHANES 2007–2018 data to develop interpretable machine learning models for predicting MetS–KSD comorbidity using dietary micronutrients and demographic variables. Random Forest and XGBoost showed the best performance under different modeling strategies, and key dietary micronutrient predictors varied according to model context.

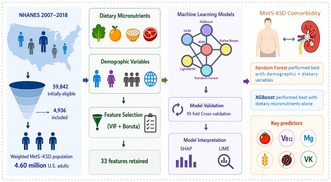

## 1 Introduction

Kidney stone disease (KSD) affects nearly 10% of the global population, and its high prevalence, together with a five‐year recurrence rate approaching 50%, presents substantial challenges for long‐term disease management and prevention (Halbritter 2023; Wang et al. 2022). In the United States, healthcare expenditures attributable to KSD exceed $10 billion annually and continue to rise in parallel with its increasing incidence, underscoring its growing public health burden (Scales Jr et al. 2012). The pathogenesis of KSD is multifactorial and complex. Accumulating epidemiological and clinical evidence indicates that metabolic syndrome (MetS), along with the chronic low‐grade inflammatory (CLGI) state it induces, substantially increases the risk of kidney stone formation (Wong et al. 2016; Bu et al. 2025; Maroufizadeh et al. 2025). Accordingly, MetS and KSD are increasingly recognized as interrelated disease entities rather than independent clinical conditions.

From a pathophysiological perspective, MetS and KSD converge on several interconnected mechanisms, most notably oxidative stress, sustained inflammatory activation, and immune dysregulation (Bargagli et al. 2025; Liu, Gao, et al. 2025). Core components of MetS—including insulin resistance, obesity, dyslipidemia, and hypertension—have been consistently associated with adverse alterations in urinary composition, such as reduced urinary pH, hypercalciuria, hyperuricosuria, and hypocitraturia (Geraghty et al. 2020). These abnormalities collectively promote a renal microenvironment conducive to crystal nucleation, growth, and retention (Hood et al. 2021). Concurrently, KSD is increasingly regarded as a manifestation of systemic metabolic dysfunction rather than a purely localized disorder of the urinary tract (Yu and Wu 2025). Within this framework, oxidative stress–driven inflammation may represent a critical mechanistic link connecting metabolic derangements to lithogenesis, thereby reinforcing the importance of integrated prevention and management strategies for MetS–KSD comorbidity. Nevertheless, factors modulating individual susceptibility within this shared pathogenic network remain incompletely elucidated.

Dietary micronutrients, including vitamins and trace elements, are known to modulate oxidative stress and inflammatory responses and may therefore influence both metabolic homeostasis and kidney stone risk (Mora‐Ortiz et al. 2024; Ojo et al. 2025; Sun et al. 2025). However, existing studies have largely focused on isolated nutrients or single disease outcomes, while conventional linear analytical approaches are inherently limited in capturing the complex, nonlinear interactions among dietary components and between nutritional exposures and metabolic phenotypes. In this context, interpretable machine learning provides a powerful framework for integrating high‐dimensional data and identifying latent risk patterns. Beyond enhancing predictive performance, such methods enable transparent feature attribution, facilitating mechanistic insight (Rajula et al. 2020). To date, population‐based investigations applying interpretable machine learning to systematically assess the contribution of dietary micronutrients to MetS–KSD comorbidity risk remain limited.

Therefore, leveraging nationally representative data from the National Health and Nutrition Examination Survey (NHANES), the present study aimed to develop and internally evaluate interpretable machine‐learning models for predicting MetS–KSD comorbidity and to systematically identify key dietary micronutrient contributors.

## 2 Materials and Methods

### 2.1 Study Population

**FIGURE 1:**
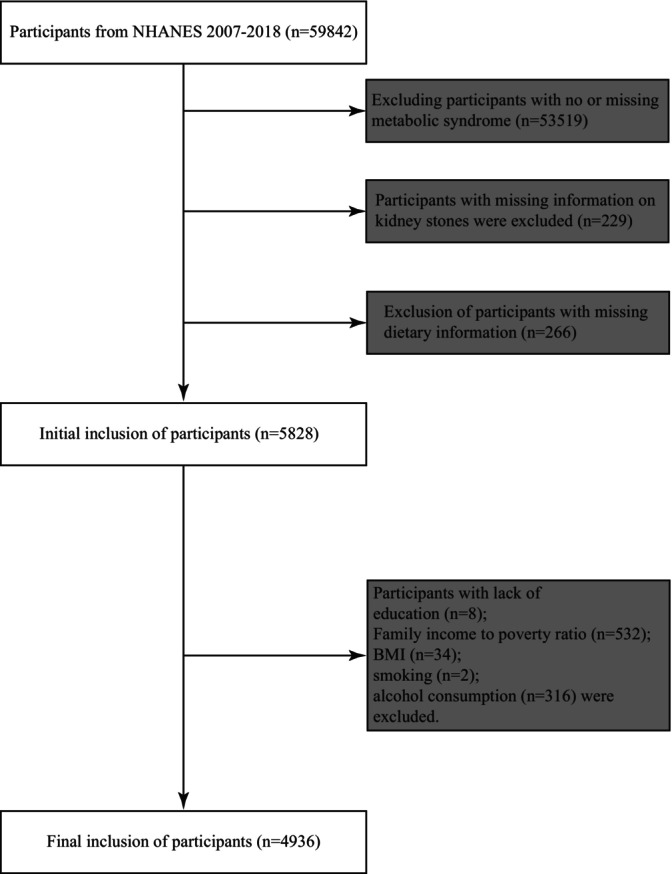
Flow chart of participant selection from NHANES 2007–2018. NHANES, National Health and Nutrition Examination Survey; BMI, body mass index.

The NHANES, administered by the National Center for Health Statistics (NCHS), is a nationally representative program designed to assess the health and nutritional status of the noninstitutionalized civilian population in the United States. This study analyzed data from NHANES survey cycles conducted between 2007 and 2018. A total of 59,842 participants were initially eligible. After applying predefined exclusion criteria, 54,906 individuals were excluded due to inability to determine MetS status or absence of MetS (*n* = 53,519), missing kidney stone diagnosis data (*n* = 229), missing dietary micronutrient information (*n* = 266), missing education data (*n* = 8), or missing key covariates—including the poverty–income ratio (PIR), body mass index (BMI), smoking status, or alcohol consumption (*n* = 884). The final analytical sample comprised 4936 participants (Figure 1).

### 2.2 Assessment of Dietary Nutrients

Dietary nutrient intake, including micronutrients (vitamins and trace elements) and selected macronutrients, was assessed using Day 1 and Day 2 dietary interview questionnaires from NHANES. Total dietary intake was collected across two nonconsecutive 24‐h dietary recall periods. The first interview was conducted in person at the Mobile Examination Center (MEC), followed by a second interview administered by telephone several days later. Both interviews were performed by trained dietary interviewers using the Automated Multiple‐Pass Method (AMPM), which systematically captures detailed information on all foods and beverages consumed.

### 2.3 Assessment of MetS and KSD

MetS was defined according to the National Cholesterol Education Program Adult Treatment Panel III (NCEP‐ATP III) criteria (Grundy et al. 2005). Participants were classified as having MetS if they met at least three of the following five components: central obesity (waist circumference ≥ 102 cm in men or ≥ 88 cm in women), hypertriglyceridemia (serum triglycerides ≥ 150 mg/dL), low high‐density lipoprotein cholesterol (serum HDL‐c < 40 mg/dL in men or < 50 mg/dL in women), hypertension (systolic blood pressure ≥ 130 mmHg or diastolic blood pressure ≥ 85 mmHg, or current use of antihypertensive medication), and hyperglycemia (fasting plasma glucose ≥ 100 mg/dL or current use of glucose‐lowering medication). Anthropometric measurements—including waist circumference, body weight, and height—were obtained at the Mobile Examination Center (MEC) by trained NHANES technicians following standardized protocols. Systolic and diastolic blood pressure values were calculated as the mean of up to four consecutive measurements obtained after a 5‐min seated rest.

Kidney stone status was ascertained using self‐reported data collected through computer‐assisted personal interviews (CAPI). Information was derived from the Kidney Conditions Questionnaire (KIQ). Participants who responded “yes” to the question “Ever had kidney stones?” were classified as having a history of KSD. This definition has been commonly adopted in prior NHANES‐based studies (Zhang, Han, et al. 2024).

### 2.4 Covariates

Sociodemographic and lifestyle variables were included as covariates, including age, sex, race/ethnicity (Mexican American, Other Hispanic, Non‐Hispanic White, Non‐Hispanic Black, and Other Race), education level, family income‐to‐poverty ratio, BMI, drinking status, and smoking status. Smoking status was categorized as non‐smokers (never smoked or quit smoking for more than 1 year) and current smokers. Drinking status was defined as non‐drinkers (fewer than 12 drinks in a lifetime) and current drinkers (at least 12 drinks per year or more than six drinking occasions in the past 12 months). BMI was calculated as weight (kg) divided by height squared (m^2^). Hypertension and diabetes were not included as covariates because they are integral components of the MetS definition, and their inclusion could introduce multicollinearity and bias the results.

### 2.5 Machine Learning Feature Preprocessing and Feature Selection

A total of 54 features were included in the analysis, comprising 49 continuous and 5 categorical variables. To mitigate multicollinearity, variance inflation factors (VIFs) were calculated, and features with a degree‐of‐freedom–adjusted VIF greater than 3 were excluded. Class imbalance was addressed using the Synthetic Minority Over‐sampling Technique (SMOTE), which was applied exclusively to the training dataset to prevent data leakage (Figure S1). All features were subsequently standardized to ensure comparable scaling during model training.

Feature selection was further performed using the Boruta algorithm, a random forest–based approach that assesses feature importance by comparing original variables with corresponding shadow features. The algorithm was iterated 500 times until convergence, and only features classified as “confirmed” were retained for subsequent model development.

### 2.6 Statistical Analyses

All statistical analyses were conducted in accordance with official NHANES analytical guidelines. Continuous variables are presented as means and standard deviations (SDs), and categorical variables as counts and weighted percentages. Group comparisons were performed using Student's *t*‐tests or chi‐square tests, as appropriate.

Six machine‐learning models were developed within the *mlr3* framework: Random Forest, Light Gradient Boosting Machine (LightGBM), K‐nearest neighbors (KNN), Naïve Bayes, support vector machine (SVM), and eXtreme Gradient Boosting (XGBoost). Hyperparameters were optimized using grid search. Model performance was evaluated using accuracy, F‐beta score, sensitivity, specificity, area under the receiver operating characteristic curve (AUC‐ROC), and area under the precision–recall curve (AUC‐PR), with AUC‐ROC designated as the primary performance metric. Repeated ten‐fold cross‐validation was applied to assess internal validity and model stability, and performance differences among models were compared using analysis of variance (*ANOVA*) and the Kruskal–Wallis *H* test.

Model interpretability was assessed using SHapley Additive exPlanations (SHAP) and Local Interpretable Model‐agnostic Explanations (LIME). SHAP provides global and local explanations by quantifying feature contributions across feature combinations, whereas LIME explains individual predictions through local surrogate models.

To further evaluate the robustness, parsimony, and practical feasibility of the proposed machine‐learning framework, a series of supplementary sensitivity analyses were additionally conducted. First, reduced‐feature models were constructed using the top 15 variables ranked according to mean absolute SHAP importance values derived from the optimal machine‐learning models under each modeling strategy. Model performance was subsequently compared with that of the original full‐feature models using multiple discrimination metrics, including AUC‐ROC, AUC‐PR, accuracy, sensitivity, and specificity. In addition, least absolute shrinkage and selection operator (LASSO) regression was performed on the 33 retained variables to further reduce feature redundancy and identify compact subsets of informative predictors. SHAP analyses were subsequently repeated on the LASSO‐selected models to assess the stability of feature importance patterns.

To further assess the influence of class imbalance correction on predictive performance, supplementary analyses were additionally performed using the original imbalanced training dataset without SMOTE processing. The performance of models trained without SMOTE processing was then compared with that of the SMOTE‐balanced models under otherwise identical training and validation procedures.

All data processing and analyses were performed using IBM SPSS Statistics (version 24.0) and R software (version 4.3.0). A two‐sided *p* value < 0.05 was considered statistically significant.

## 3 Results

### 3.1 Participant Characteristics by MetS–KSD Comorbidity

Baseline characteristics according to the presence of MetS–KSD comorbidity are shown in Table 1. The analytical sample comprised 4936 participants from the NHANES 2007–2018 cycles, representing an estimated 33,743,271 community‐dwelling U.S. adults after applying sampling weights. The overall mean age was 53.43 years (SD = 15.38), and women accounted for 51.0% of the study population. After weighting, approximately 4,597,435 individuals were classified as having MetS–KSD comorbidity, with a mean age of 55.97 years (SD = 14.07). Compared with participants without comorbidity, those with MetS–KSD comorbidity exhibited significantly lower intakes of protein, niacin, phosphorus, magnesium, and selenium (all *p* < 0.05).

**TABLE 1:** Baseline characteristics of participants by MetS–KSD comorbidity status.

Characteristic	*N*	Overall (*N* = 33,743,271)	No MetS–KSD comorbidity (*N* = 29,145,837)	MetS–KSD comorbidity (*N* = 4,597,435)	*p*
Age (years)^a^, Mean ± SD	4936	53.43 ± (15.38)	53.03 ± (15.54)	55.97 ± (14.07)	< 0.001
Sex^b^, *n* (%)					
Female	4936	2602 (51%)	2290 (52%)	312 (49%)	0.318
Male		2334 (49%)	1994 (48%)	340 (51%)	
Race/ethnicity^b^, *n* (%)					
Mexican American	4936	863 (9.2%)	764 (9.6%)	99 (6.8%)	< 0.001
Other Hispanic		540 (5.7%)	473 (5.8%)	67 (4.4%)	
Non‐Hispanic White		2313 (70%)	1932 (69%)	381 (78%)	
Non‐Hispanic Black		853 (8.9%)	793 (9.7%)	60 (3.8%)	
Other Race		367 (6.2%)	322 (6.1%)	45 (6.8%)	
Education^b^, *n* (%)					
Less Than 9th	4936	600 (6.1%)	532 (6.2%)	68 (5.0%)	0.541
9–11th		796 (12%)	689 (12%)	107 (12%)	
High School		1231 (27%)	1085 (28%)	146 (24%)	
Some College		1513 (33%)	1279 (33%)	234 (36%)	
College Graduate		796 (21%)	699 (21%)	97 (22%)	
Family income to poverty ratio^a^, Mean ± SD	4936	2.78 ± (1.62)	2.78 ± (1.63)	2.78 ± (1.55)	0.936
BMI^a^, Mean ± SD	4936	33.61 ± (6.84)	33.50 ± (6.80)	34.31 ± (7.07)	0.112
Smoking status^b^, *n* (%)					
No	4936	3944 (80%)	3414 (79%)	530 (84%)	0.119
Yes		992 (20%)	870 (21%)	122 (16%)	
Drinking status^b^, *n* (%)					
No	4936	757 (12%)	660 (11%)	97 (15%)	0.047
Yes		4179 (88%)	3624 (89%)	555 (85%)	
Energy^a^, Mean ± SD	4936	2127.75 ± (975.81)	2133.51 ± (985.71)	2091.25 ± (910.43)	0.630
Protein^a^, Mean ± SD	4936	82.08 ± (41.48)	82.83 ± (41.96)	77.34 ± (38.00)	0.022
Carbohydrate^a^, Mean ± SD	4936	252.76 ± (125.12)	253.23 ± (126.70)	249.75 ± (114.68)	0.856
Total Sugar^a^, Mean ± SD	4936	112.76 ± (81.27)	112.69 ± (81.74)	113.22 ± (78.35)	0.692
Dietary fiber^a^, Mean ± SD	4936	16.38 ± (9.78)	16.38 ± (9.83)	16.38 ± (9.41)	0.811
Total Fat^a^, Mean ± SD	4936	83.90 ± (46.92)	83.68 ± (46.96)	85.29 ± (46.73)	0.468
Saturated fatty acids^a^, Mean ± SD	4936	27.50 ± (16.96)	27.44 ± (17.07)	27.88 ± (16.24)	0.352
Monounsaturated fatty acids^a^, Mean ± SD	4936	29.63 ± (17.58)	29.53 ± (17.50)	30.24 ± (18.08)	0.560
Polyunsaturated fatty acids^a^, Mean ± SD	4936	19.11 ± (12.48)	19.06 ± (12.49)	19.44 ± (12.48)	0.639
Cholesterol^a^, Mean ± SD	4936	304.10 ± (243.54)	304.65 ± (240.94)	300.61 ± (259.59)	0.328
Vitamin E as alpha‐tocopherol^a^, Mean ± SD	4936	8.34 ± (6.26)	8.36 ± (6.31)	8.25 ± (5.90)	0.909
Alpha‐tocopherol^a^, Mean ± SD	4936	0.65 ± (3.14)	0.67 ± (3.27)	0.49 ± (2.19)	0.903
Retinol^a^, Mean ± SD	4936	413.64 ± (373.47)	418.50 ± (385.62)	382.82 ± (282.89)	0.581
Vitamin A^a^, Mean ± SD	4936	597.91 ± (517.12)	604.02 ± (533.04)	559.18 ± (399.87)	0.779
Alpha‐carotene^a^, Mean ± SD	4936	348.44 ± (878.53)	349.66 ± (877.08)	340.70 ± (888.29)	0.816
Beta‐carotene^a^, Mean ± SD	4936	2001.29 ± (3569.49)	2014.98 ± (3638.49)	1914.48 ± (3097.52)	0.634
Beta‐cryptoxanthin^a^, Mean ± SD	4936	79.36 ± (298.66)	79.72 ± (314.95)	77.08 ± (160.80)	0.389
Lycopene^a^, Mean ± SD	4936	5335.15 ± (9555.19)	5328.53 ± (9595.66)	5377.11 ± (9301.58)	0.397
Lutein + zeaxanthin^a^, Mean ± SD	4936	1335.86 ± (2511.78)	1343.83 ± (2596.26)	1285.35 ± (1891.12)	0.577
Thiamin (Vitamin B1)^a^, Mean ± SD	4936	1.60 ± (0.87)	1.60 ± (0.88)	1.55 ± (0.79)	0.497
Riboflavin (Vitamin B2)^a^, Mean ± SD	4936	2.14 ± (1.27)	2.17 ± (1.31)	1.99 ± (1.03)	0.105
Niacin^a^, Mean ± SD	4936	25.14 ± (15.52)	25.42 ± (15.77)	23.41 ± (13.71)	0.049
Vitamin B6^a^, Mean ± SD	4936	2.03 ± (1.83)	2.05 ± (1.90)	1.88 ± (1.22)	0.243
Total folate^a^, Mean ± SD	4936	389.54 ± (239.00)	390.89 ± (240.13)	380.97 ± (231.68)	0.585
Folic acid^a^, Mean ± SD	4936	177.17 ± (173.08)	177.93 ± (174.07)	172.36 ± (166.75)	0.692
Food folate^a^, Mean ± SD	4936	212.42 ± (130.31)	213.03 ± (130.39)	208.54 ± (129.77)	0.428
Folate (DFE)^a^, Mean ± SD	4936	513.42 ± (347.65)	515.31 ± (349.53)	501.44 ± (335.46)	0.619
Total choline^a^, Mean ± SD	4936	336.15 ± (201.29)	338.75 ± (203.14)	319.68 ± (188.45)	0.109
Vitamin B12^a^, Mean ± SD	4936	5.07 ± (5.29)	5.12 ± (5.45)	4.79 ± (4.17)	0.880
Added vitamin B12^a^, Mean ± SD	4936	0.90 ± (2.72)	0.90 ± (2.79)	0.88 ± (2.29)	0.550
Vitamin C^a^, Mean ± SD	4936	73.67 ± (81.93)	74.16 ± (81.70)	70.57 ± (83.40)	0.519
Vitamin K^a^, Mean ± SD	4936	104.45 ± (134.67)	104.30 ± (137.10)	105.42 ± (118.18)	0.912
Calcium^a^, Mean ± SD	4936	955.18 ± (587.50)	964.38 ± (595.23)	896.85 ± (532.67)	0.066
Phosphorus^a^, Mean ± SD	4936	1379.16 ± (673.28)	1391.53 ± (682.51)	1300.77 ± (606.17)	0.036
Magnesium^a^, Mean ± SD	4936	292.66 ± (146.11)	295.59 ± (148.44)	274.05 ± (128.89)	0.040
Iron^a^, Mean ± SD	4936	14.67 ± (8.42)	14.71 ± (8.54)	14.43 ± (7.58)	0.998
Zinc^a^, Mean ± SD	4936	11.54 ± (7.84)	11.67 ± (8.12)	10.76 ± (5.65)	0.321
Copper^a^, Mean ± SD	4936	1.22 ± (0.72)	1.23 ± (0.75)	1.16 ± (0.56)	0.276
Sodium^a^, Mean ± SD	4936	3580.21 ± (1857.06)	3585.05 ± (1852.07)	3549.48 ± (1889.54)	0.562
Potassium^a^, Mean ± SD	4936	2638.74 ± (1231.52)	2661.92 ± (1254.78)	2491.80 ± (1061.50)	0.115
Selenium^a^, Mean ± SD	4936	115.07 ± (62.65)	116.30 ± (63.24)	107.29 ± (58.24)	0.008
Caffeine^a^, Mean ± SD	4936	190.02 ± (234.06)	193.46 ± (239.62)	168.18 ± (193.91)	0.147
Theobrominea, Mean ± SD	4936	36.54 ± (76.02)	36.44 ± (78.40)	37.19 ± (58.73)	< 0.001
Alcohol^a^, Mean ± SD	4936	8.45 ± (25.46)	8.83 ± (26.42)	6.06 ± (18.01)	0.118
Moisture^a^, Mean ± SD	4936	3069.26 ± (1555.59)	3084.47 ± (1575.19)	2972.84 ± (1422.44)	0.302

*Note:* Values are presented as weighted means ± standard deviations or weighted percentages.

Abbreviation: SD, standard deviation.

^a^ Student's *t*‐test.

^b^ Chi‐square test.

### 3.2 Feature Selection for Machine‐Learning Models

VIF analysis was performed to assess multicollinearity among candidate features, with degrees‐of‐freedom–adjusted VIF values greater than 3 indicating collinearity. A total of 21 features were identified as collinear and excluded from subsequent analyses. The remaining variables were further evaluated using the Boruta algorithm, and all retained features were classified as having confirmed contributions to MetS–KSD comorbidity. Detailed results of VIF assessment, Boruta‐based feature selection, and the evolution of Z‐scores across Boruta iterations are presented in Figure S2. Ultimately, 33 features were retained for model development, including 25 dietary micronutrients and 8 demographic variables.

### 3.3 Construction and Evaluation of Machine‐Learning Models

**FIGURE 2:**
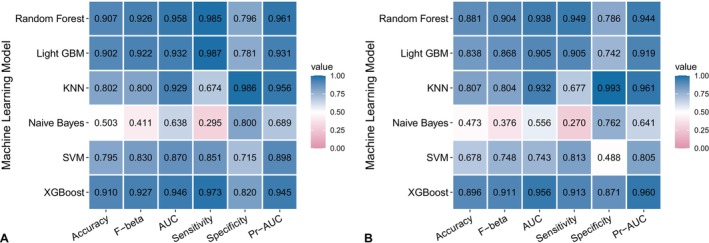
Comparative heatmap of machine learning model performance under two modeling strategies. (A) Models incorporating demographic variables and dietary micronutrients. (B) Models using dietary micronutrients alone.

The predictive performance of six machine‐learning models (Random Forest, LightGBM, KNN, Naïve Bayes, SVM, and XGBoost) across multiple discrimination metrics—including AUC‐ROC, AUC‐PR, accuracy, F‐beta score, sensitivity, and specificity—was visualized using heatmaps under two modeling strategies (Figure 2): demographic variables combined with dietary micronutrients and dietary micronutrients alone. The full performance metrics of the primary SMOTE‐balanced models are summarized in Tables S1 and S2.

**FIGURE 3:**
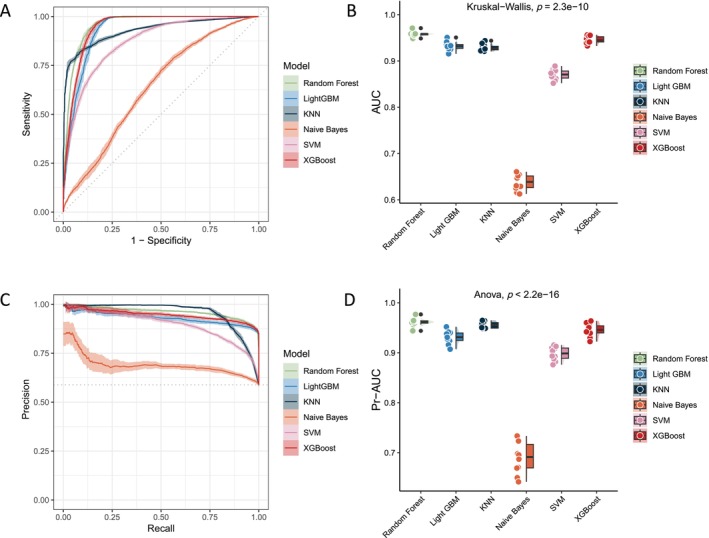
ROC and precision–recall performance of machine learning models incorporating demographic variables and dietary micronutrients. (A) ROC curves for MetS–KSD comorbidity prediction. (B) Distribution of AUC‐ROC values. (C) Precision–recall curves. (D) Distribution of AUC‐PR values.

When demographic variables were included, the Random Forest model demonstrated the strongest overall performance across discrimination metrics, achieving an AUC‐ROC of 0.958 and an AUC‐PR of 0.961 (Figure 3), together with high accuracy (0.907) and sensitivity (0.985). XGBoost and LightGBM showed slightly lower but comparable performance (AUC‐ROC: 0.946 and 0.932, respectively), whereas KNN and SVM exhibited moderate discriminative ability. Naïve Bayes consistently performed worst among the evaluated models. Performance differences across models were statistically significant for AUC‐ROC and AUC‐PR, and similar trends were also observed for accuracy, F‐beta score, sensitivity, and specificity (all *p* < 0.001).

**FIGURE 4:**
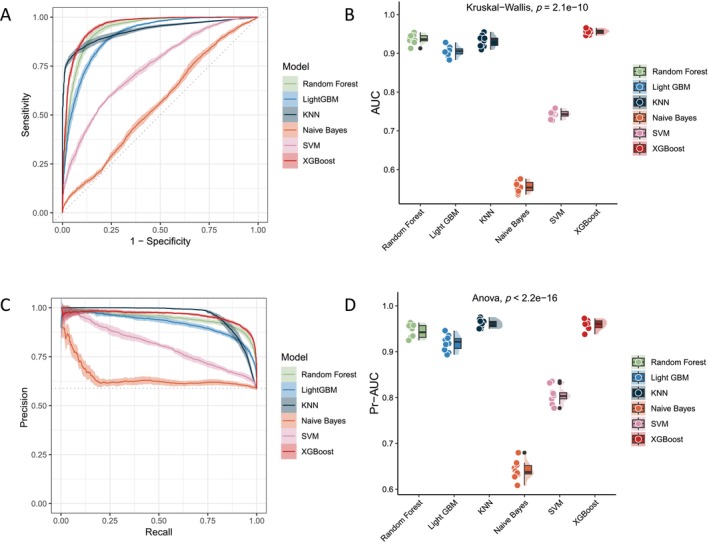
ROC and precision–recall performance of machine learning models using dietary micronutrients alone. (A) ROC curves for MetS–KSD comorbidity prediction. (B) Distribution of AUC‐ROC values. (C) Precision–recall curves. (D) Distribution of AUC‐PR values.

When models were constructed using dietary micronutrients alone, XGBoost exhibited the best overall performance, with an AUC‐ROC of 0.956 and an AUC‐PR of 0.960 (Figure 4), along with balanced accuracy (0.896) and specificity (0.871). Random Forest and LightGBM followed closely (AUC‐ROC: 0.938 and 0.905, respectively), while KNN and SVM again showed moderate performance and Naïve Bayes remained the weakest performer. Similar performance patterns were observed for accuracy, F‐beta score, sensitivity, and specificity, with statistically significant differences among models under the dietary micronutrient‐only strategy (all *p* < 0.001).

### 3.4 Interpretation of Dietary Micronutrients Importance Using SHAP and LIME

**FIGURE 5:**
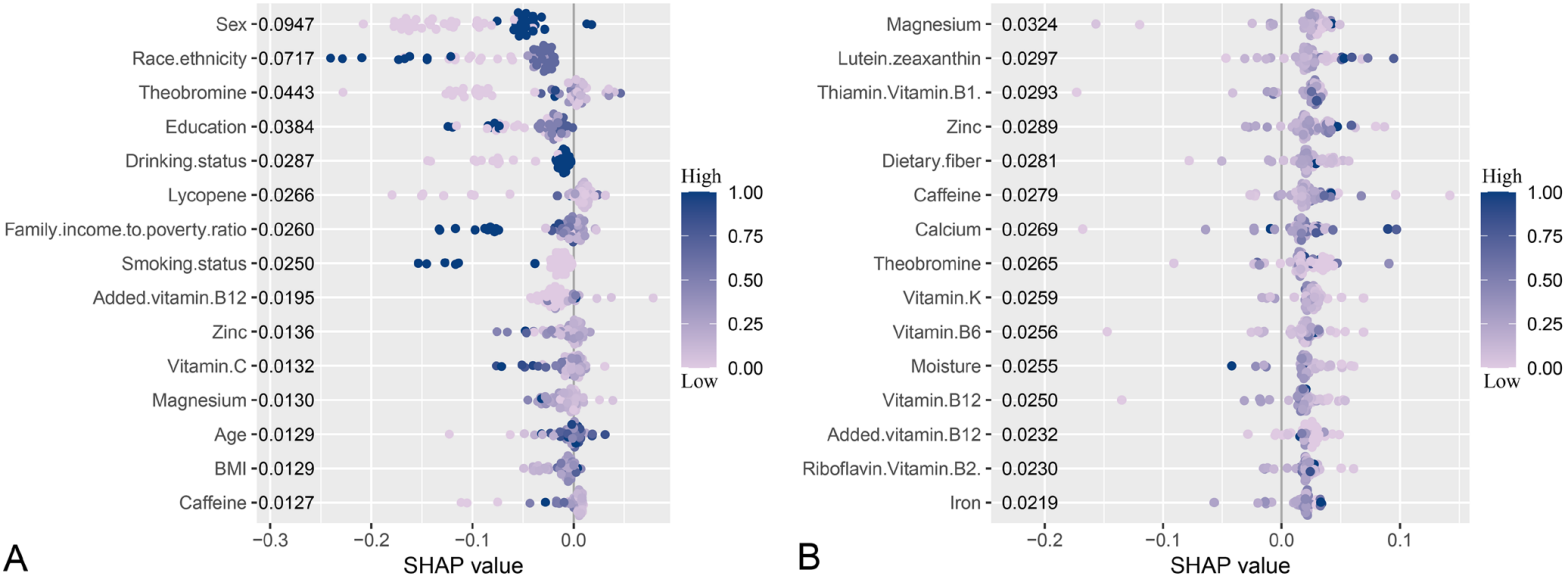
Global SHAP importance of the top 15 features under two modeling strategies. (A) SHAP summary plot for the Random Forest model incorporating demographic variables and dietary micronutrients. (B) SHAP summary plot for the XGBoost model using dietary micronutrients alone. Each point represents an individual participant, with color indicating feature value and horizontal position indicating the SHAP value.

To elucidate the contributions of individual features to model predictions under different modeling strategies, SHAP and LIME analyses were performed. Figure 5 presents SHAP summary plots of the top 15 features under the two modeling strategies: the Random Forest model incorporating both demographic characteristics and dietary micronutrients (Figure 5A) and the XGBoost model using dietary micronutrients alone (Figure 5B), thereby providing an overview of population‐level feature contributions to MetS–KSD comorbidity risk. SHAP values were used to quantify the direction and magnitude of feature contributions to model output. In the combined model, lycopene (SHAP = 0.0266), added vitamin B12 (0.0195), zinc (0.0136), vitamin C (0.0132), and magnesium (0.0130) emerged as the most influential dietary contributors, indicating their prominent roles in risk stratification when demographic context was considered. When the model was restricted to dietary micronutrients alone, a distinct yet partially overlapping importance pattern was observed, characterized by greater contributions from nutrients related to mineral balance and dietary structure, such as magnesium, dietary fiber, theobromine, vitamin K, and vitamin B6. Corresponding rankings based on mean absolute SHAP values are shown in Figure S3. This contrast highlights the context‐dependent nature of dietary micronutrient importance in predicting MetS–KSD comorbidity.

Force plots and waterfall plots were used to provide case‐level explanations for the Random Forest and XGBoost models, illustrating how aggregated feature contributions shifted the predicted probability for representative participants relative to the baseline output. In the model including demographic and dietary variables, the baseline predicted probability of no comorbidity was 0.353 and increased to 0.839 after accounting for feature contributions (Figure S4). When only dietary micronutrients were considered, the baseline probability of no comorbidity was 0.600 and increased markedly after feature aggregation (Figure S5). Interaction dependence plots further illustrated the nonlinear relationships between key dietary micronutrients and their SHAP contributions under different modeling strategies (Figure S6).

To further characterize individualized predictions, LIME was applied for local interpretation of the Random Forest and XGBoost models. When demographic variables were included, the predicted probability of no comorbidity for the illustrative case was 0.94, with potassium (3527–3656), copper (1.462–1.600), vitamin D (6.70–6.80), and sodium (2458–3618) identified as contributing features in the local explanation (Figure S7). When only dietary micronutrients were considered, the predicted probability of no comorbidity for the illustrative case increased markedly, and the local explanation highlighted alpha‐tocopherol ≤ 3.47, added vitamin B12 ≤ 2.08, vitamin K (57.2–91.6), riboflavin (vitamin B2) (2.07–3.01), caffeine ≤ 80.1, and vitamin E (6.70–9.49) as contributing features within the local neighborhood of that observation (Figure S8).

**FIGURE 6:**
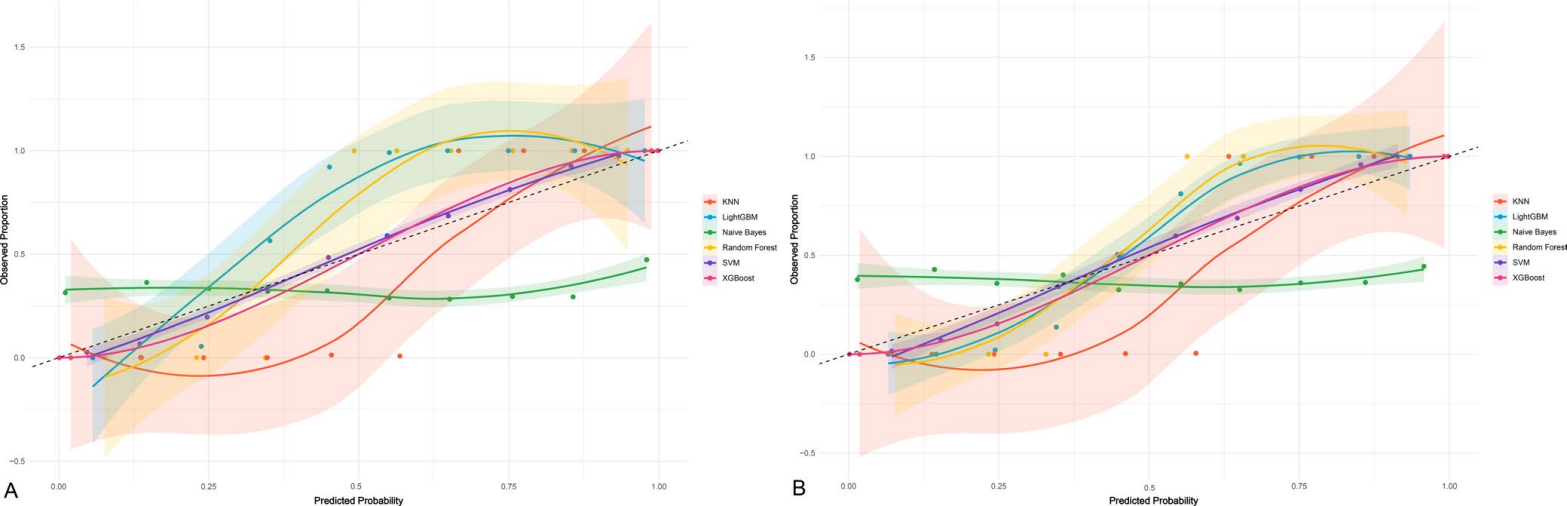
Calibration curves for machine learning models predicting MetS–KSD comorbidity under two modeling strategies. (A) Models incorporating demographic variables and dietary micronutrients. (B) Models using dietary micronutrients alone. The diagonal line indicates perfect calibration.

Calibration curves were used to assess model calibration (Figure 6). Good agreement between predicted and observed outcomes was observed for models incorporating both demographic and dietary micronutrients as well as for models including dietary micronutrients alone, indicating satisfactory calibration across modeling strategies.

### 3.5 Sensitivity Analyses

To further evaluate the robustness, parsimony, and practical feasibility of the proposed machine‐learning framework, a series of supplementary sensitivity analyses were additionally conducted. First, reduced‐feature models were reconstructed using the top 15 variables ranked according to mean absolute SHAP importance values derived from the optimal machine‐learning models under each modeling strategy. Compared with the original full‐feature models, the reduced‐feature models maintained broadly comparable discrimination performance while substantially reducing overall model complexity. In the combined demographic and dietary micronutrient model, the Random Forest classifier achieved an AUC‐ROC of 0.937 and an AUC‐PR of 0.936. In the dietary micronutrient‐only model, XGBoost achieved an AUC‐ROC of 0.929 and an AUC‐PR of 0.943 (Table S3 and Figure S9).

In addition, LASSO regression was further performed on the 33 retained variables to reduce feature redundancy and identify compact subsets of informative predictors (Figure S10). Machine‐learning models reconstructed using the LASSO‐selected variables demonstrated comparable predictive performance to the original full‐feature models. Subsequent SHAP analyses based on the LASSO‐selected features demonstrated that the major dietary micronutrients identified as important contributors were broadly consistent with those highlighted in the primary analyses, supporting the relative robustness and stability of the identified feature importance patterns (Table S4 and Figure S11).

To further assess the influence of class imbalance correction on predictive performance, supplementary analyses were performed using the original imbalanced training dataset without SMOTE processing under otherwise identical training and validation procedures. Models trained without SMOTE showed inferior overall discrimination performance and markedly imbalanced classification profiles. Specifically, the best‐performing models achieved AUC‐ROC values of only 0.565 in the dietary micronutrient‐only strategy and 0.511 in the combined demographic and dietary strategy, accompanied by extremely low specificity in several models. By contrast, after SMOTE balancing was applied to the training dataset, model performance improved substantially, with Random Forest achieving an AUC‐ROC of 0.958 and an AUC‐PR of 0.961 in the combined strategy, and XGBoost achieving an AUC‐ROC of 0.956 and an AUC‐PR of 0.960 in the dietary micronutrient‐only strategy. These findings indicate that SMOTE effectively mitigated the adverse influence of class imbalance on MetS–KSD comorbidity prediction by improving overall discrimination performance and classification balance. The performance metrics of models trained without SMOTE processing are presented in Table S5, and graphical comparisons with the primary SMOTE‐balanced models are shown in Figure S12.

## 4 Discussion

In this nationally representative analysis of U.S. adults, we applied interpretable machine‐learning approaches to examine associations between dietary micronutrient intake and MetS–KSD comorbidity using NHANES data from 2007 to 2018. Across the evaluated algorithms, model performance was robust and consistent, with Random Forest achieving optimal discrimination when demographic and dietary variables were jointly modeled, whereas XGBoost performed best when dietary micronutrients were considered alone. To enhance interpretability, we employed SHAP and LIME, which provide complementary insights by characterizing feature contributions at both population and individual levels, thereby elucidating how dietary micronutrients inform risk prediction across different modeling contexts.

Converging evidence indicates that MetS is closely linked to kidney stone formation, supporting the paradigm that KSD often arises within a broader context of systemic metabolic dysfunction rather than as an isolated urological disorder (Bu et al. 2025; Maroufizadeh et al. 2025; Bargagli et al. 2025; Liu, Gao, et al. 2025). In particular, diabetes, obesity, and related metabolic abnormalities may promote oxidative stress and CLGI (Mirahmadi et al. 2023; Islam et al. 2025), processes that have been implicated in increased renal tubular injury and the facilitation of crystal retention (Lin et al. 2024). Persistent oxidative and inflammatory activation has been implicated in renal tubular vulnerability and altered host–crystal interactions.

Previous studies have shown that individuals with type 2 diabetes often exhibit characteristic urinary alterations, including increased oxalate excretion and persistently reduced urinary pH (Eisner et al. 2010), thereby creating a physicochemical milieu permissive to crystal formation. In parallel, calcium oxalate crystals themselves can induce oxidative stress responses, increasing reactive oxygen species (ROS) production within tubular epithelial cells, which may exacerbate tubular injury and enhance crystal adhesion and retention (Kumar et al. 2023). Disturbances in glucose metabolism and the accompanying insulin resistance further contribute to urinary acidification by reducing urinary citrate availability, thereby weakening endogenous crystallization inhibition and facilitating crystal persistence (Strohmaier et al. 2012; Cupisti et al. 2007). Obesity, a core component of MetS, may further amplify lithogenic susceptibility through sustained oxidative stress and CLGI (Ghowsi et al. 2021). Obese individuals commonly exhibit chronic inflammatory activation, with altered levels of inflammatory mediators—such as tumor necrosis factor (TNF) and interleukin family cytokines—detectable in renal tissue and urine (Ye et al. 2023; Lovegrove et al. 2023). These inflammatory and metabolic disturbances may impair tubular epithelial function, alter urinary calcium handling, and promote calcium salt deposition, collectively shaping a renal environment permissive to crystallization. Collectively, the cluster of metabolic abnormalities defining MetS is associated with an adverse urinary milieu, including increased excretion of urinary calcium, uric acid, and oxalate, reduced citrate excretion, and lower urinary pH, which—under the combined influence of oxidative stress and CLGI—may continuously shape a renal microenvironment conducive to crystal formation, growth, and retention.

Within the broader context of metabolic dysregulation, oxidative stress, and inflammation, specific nutrients may be better conceptualized as modifiers of MetS–KSD comorbidity risk rather than direct causal determinants. At the population level, consistent associations were observed between MetS–KSD comorbidity and lower intakes of several dietary micronutrients, including protein, niacin, phosphorus, magnesium, and selenium. Reduced protein intake may not simply reflect inadequate total protein consumption, but rather a shift in protein intake patterns, particularly insufficient intake of plant‐derived protein–rich dietary patterns (Bu et al. 2025; Ghazvineh et al. 2025). Such structural changes in protein intake may weaken urinary buffering capacity—manifested by reduced citrate availability and lower urinary pH—and diminish crystallization‐inhibitory effects (Aleksandrova et al. 2021). These alterations are often accompanied by overall poorer diet quality and are closely linked to disordered energy metabolism and impaired antioxidant defense. Niacin (vitamin B3), as an essential precursor of NAD^+^, plays a central role in cellular energy metabolism and redox homeostasis. Insufficient niacin intake may compromise mitochondrial function and antioxidant capacity, thereby exacerbating oxidative stress in individuals with MetS–KSD comorbidity (Elhassan et al. 2017). Magnesium deficiency may simultaneously reduce urinary crystallization inhibition and aggravate insulin resistance and inflammatory burden, creating a renal microenvironment more permissive to stone formation in metabolically vulnerable individuals (Zhao et al. 2025). Phosphorus intake is closely tied to calcium–phosphate homeostasis and may reflect overall dietary mineral balance; insufficient intake may therefore be associated with unfavorable urinary calcium handling (Zhang, Lou, et al. 2024). Selenium, an essential component of antioxidant selenoproteins, is critical for redox regulation and inflammatory control (Hariharan and Dharmaraj 2020). Reduced selenium intake may weaken antioxidant defenses and increase renal tubular susceptibility to oxidative injury under conditions of chronic metabolic stress (Pei et al. 2023). Collectively, these nutrients constitute an interconnected regulatory network supporting metabolic stability, antioxidant defense, and mineral homeostasis. Inadequate intake across this network may amplify oxidative stress and inflammatory signaling, thereby lowering renal resistance to crystallization and facilitating the development and progression of kidney stone disease in the setting of metabolic syndrome.

Given the multifactorial and nonlinear nature of the metabolic and nutritional pathways underlying MetS–KSD comorbidity, we implemented multiple machine‐learning algorithms and benchmarked them a priori to align model selection with feature structure and analytical objectives. Compared with traditional regression‐based approaches, machine‐learning methods can capture complex, nonlinear interactions among dietary micronutrients, metabolic traits, and demographic factors without relying on prespecified distributional assumptions, while ensemble‐based algorithms facilitate data‐driven feature prioritization and reduce subjectivity in variable selection (Lin et al. 2023; Zhang et al. 2025a). Model performance differed by feature composition: when demographic characteristics were jointly modeled with dietary micronutrients, Random Forest showed superior discrimination (AUC‐ROC = 0.958; AUC‐PR = 0.961), with XGBoost and LightGBM performing similarly but slightly lower. When dietary micronutrients were modeled alone, XGBoost achieved optimal performance (AUC‐ROC = 0.956; AUC‐PR = 0.960), followed by Random Forest and LightGBM. This divergence likely reflects differences between bagging and boosting: Random Forest is well suited to heterogeneous epidemiological data because it is robust to multicollinearity, noise, and nonlinear interactions across mixed feature types (Stonier et al. 2023), whereas XGBoost can capture subtle, high‐dimensional nonlinear relationships within continuous variables, which may be advantageous in a micronutrient‐only feature space (Kumari et al. 2025). This benchmarking strategy supports the interpretation that observed performance differences reflect underlying data structure rather than post hoc model selection. Nevertheless, because these findings are based on internal validation only, the relatively high discriminative performance should be interpreted cautiously until confirmed in independent external cohorts.

To enhance model interpretability, we employed SHAP and LIME to elucidate how key features influence predictions (Zhu et al. 2025). SHAP offers a game‐theory‐based framework to quantify each feature's marginal contribution, enabling both global importance rankings and visualizations of directional effects on predictions (Xu et al. 2025; Netayawijit et al. 2025). In parallel, LIME generates locally faithful explanations by approximating model behavior around individual instances, thereby revealing case‐level drivers of predictions (Guattery et al. 2025). Together, these methods provide complementary insights—SHAP highlights population‐level feature relevance, while LIME uncovers individualized decision pathways—reducing the limitations inherent in relying on a single interpretability approach (Liu, Wu, et al. 2025).

A key finding of this study is that the dietary micronutrients highlighted by SHAP varied depending on whether demographic variables were included in the model. This pattern does not reflect a methodological limitation but rather underscores the context‐dependent nature of nutritional effects in MetS–KSD comorbidity. Nutrients do not exert fixed or uniform influences; instead, their predictive relevance emerges through interaction with the underlying metabolic and inflammatory milieu, approximated here by demographic and clinical variables (Qi et al. 2025). When demographic factors were incorporated, the model accounted for baseline metabolic vulnerability characterized by heightened oxidative stress and inflammatory activity. Under these conditions, micronutrients with more direct redox‐modulating or anti‐inflammatory properties—such as lycopene (Bai et al. 2023) and added vitamin B12 (Mathew et al. 2024)—emerged as prominent contributors, suggesting that their relevance may be amplified in individuals with pre‐existing metabolic stress. In contrast, when the model relied solely on dietary inputs, nutrients such as dietary fiber, theobromine, and vitamin K became more influential. These factors are more closely linked to upstream regulatory pathways, including gut microbiota modulation, inflammatory signaling, and mineral metabolism, and may therefore convey broader dietary patterns associated with reduced metabolic burden (Zhao et al. 2022; Gao et al. 2022; Zhang et al. 2025b). The consistent importance of magnesium across modeling contexts is particularly noteworthy. This stability suggests it occupies a unique nodal position in the pathophysiology, simultaneously influencing insulin receptor sensitivity (Wan Nik et al. 2023), NF‐κB‐mediated inflammation (Sadikan et al. 2025), and direct competition with calcium in crystal lattice formation (Zhao et al. 2025). This multi‐target engagement may render its protective effect more robust to variations in baseline metabolic status, making it a broad‐spectrum metabolic stabilizer. LIME analyses extended these observations to the individual level, revealing that the features driving low‐risk predictions for the same representative participant differed markedly depending on the available information. When demographic context was included, nutrients such as potassium, copper, and vitamin D were most influential, indicating interactions with known metabolic background (Passador et al. 2018). In the absence of such context, the model emphasized nutrients related to systemic antioxidant and anti‐inflammatory capacity, including alpha‐tocopherol (Rouaki et al. 2013) and vitamin K (Zhang et al. 2025b). Together, these findings illustrate that nutrition‐related risk is not governed by a fixed set of “key nutrients,” but by dynamic interactions between dietary status and the broader metabolic environment. In summary, SHAP and LIME analyses converge on a coherent interpretation: dietary micronutrients in MetS–KSD comorbidity primarily function as context‐sensitive modulators of metabolic resilience. Their importance is revealed conditionally, depending on the physiological background in which they operate, reinforcing the concept that nutritional effects are embedded within, rather than independent of, systemic metabolic and inflammatory networks. Accordingly, the present results should not be construed as evidence that increasing intake of specific micronutrients alone is sufficient to prevent MetS–KSD comorbidity. Rather, they highlight the potential importance of maintaining adequate and balanced micronutrient status as part of broader metabolic risk management. Within a systemic metabolic disorder characterized by oxidative stress, inflammation, and adverse urinary profiles, nutritional status may influence the threshold at which these perturbations translate into clinically manifest stone disease (Boyd et al. 2018).

This study offers several noteworthy strengths that advance the methodology and interpretation of diet–disease relationships in comorbid conditions. To our knowledge, it is the first to develop and internally evaluate an interpretable machine‐learning framework for predicting MetS–KSD comorbidity using nationally representative dietary micronutrient data. The analysis is grounded in nationally representative NHANES data and supported by rigorous algorithmic comparison, demonstrating stable internal predictive performance within a large population‐based dataset. By integrating demographic and lifestyle variables, the models achieved improved predictive performance and enhanced interpretability, moving beyond fixed risk factors alone. The joint application of SHAP and LIME provides transparent, multi‐level interpretation—revealing population‐level feature importance alongside individualized decision pathways. Ultimately, by framing dietary micronutrients as context‐dependent metabolic modifiers rather than isolated causal agents, this work provides a systems‐level perspective that can inform future nutritional, mechanistic, and translational research on MetS–KSD and other metabolically linked diseases.

From a practical perspective, the present framework may serve as a supportive tool for nutritional risk stratification in individuals with metabolic vulnerability, particularly in settings where demographic and dietary information can be routinely collected. Rather than functioning as a stand‐alone diagnostic system, the model may help identify individuals with MetS who are at higher predicted risk of KSD and who may benefit from closer metabolic profiling, individualized dietary counseling, and earlier preventive monitoring. In this context, interpretable machine learning may offer transparent and explainable risk information to support early risk identification and targeted prevention strategies for MetS–KSD comorbidity.

Several limitations of this study should be acknowledged. First, the cross‐sectional nature of the NHANES data precludes causal inference between dietary micronutrient intake and MetS–KSD comorbidity; the observed relationships should therefore be interpreted as associations rather than mechanistic evidence. In addition, kidney stone history was self‐reported, which may introduce recall bias or misclassification, although previous studies have suggested acceptable validity and any such misclassification is likely to be non‐differential with respect to dietary intake. Second, although SMOTE was applied exclusively to the training set and model performance was assessed using repeated 10‐fold cross‐validation, the present study did not include an independent external validation cohort. Although the ensemble‐based machine‐learning models demonstrated strong discrimination performance, the possibility of mild overfitting cannot be completely excluded, particularly given the relatively high predictive performance achieved by the Random Forest and XGBoost models. Nevertheless, several methodological strategies were implemented to reduce overfitting risk, including repeated 10‐fold cross‐validation, restriction of SMOTE balancing to training folds only, reduced‐feature sensitivity analyses, and additional LASSO‐based feature selection. Furthermore, simplified models constructed using top SHAP‐ranked variables maintained broadly comparable predictive performance despite substantially reduced model complexity, further supporting the internal robustness of the proposed framework. Accordingly, the relatively high discriminative performance observed in this study should be interpreted with caution, given the potential risk of overfitting. Third, the stringent exclusion criteria required to ensure complete dietary and covariate data may have introduced selection bias. Moreover, although NHANES includes multiple racial and ethnic subgroups, it still reflects a single U.S.‐based population context. Differences in dietary patterns, metabolic profiles, social environments, and healthcare systems may therefore limit the generalizability of our findings to other populations. Finally, although the final model was derived through structured feature preprocessing and selection procedures, it still retained multiple variables, which may limit direct implementation in routine practice. Future studies should further optimize and simplify the model to enhance its clinical applicability and translational potential. In addition, while SHAP and LIME improve interpretability, they reflect model‐based feature importance rather than biological causation. Further longitudinal and experimental studies, together with external validation in more diverse populations, are needed to confirm these findings and further evaluate the applicability of the proposed framework.

## 5 Conclusion

In conclusion, we developed and internally evaluated interpretable machine‐learning models to predict MetS–KSD comorbidity using nationally representative dietary micronutrient data. Among the evaluated algorithms, ensemble‐based models showed the strongest discriminative performance, with optimal model selection varying according to feature composition. Interpretable analyses further clarified the contribution of dietary micronutrient patterns to comorbidity risk within a metabolic–inflammatory context. These findings suggest that interpretable machine learning may provide a useful framework for nutritional risk stratification in MetS–KSD comorbidity. External validation in independent populations is still needed before broader application.

## Author Contributions

**Haibo Qin:** methodology, investigation, validation, writing – review and editing. **Guanwei Wu:** conceptualization, methodology, writing – original draft. **Jie Zheng:** project administration, formal analysis. **Junfeng Yao:** methodology, software, data curation. **Yingqing Liu:** validation, project administration, resources. **Jiawei Wang:** visualization, software, formal analysis. **Zongyao Hao:** validation, visualization. **Lingsong Tao:** visualization, project administration, supervision.

## Funding

This work was supported by the Key Project of the Wuhu Science and Technology Bureau (2023jc32) and the Research Project of the Affiliated Wuhu Hospital of East China Normal University (2024A04).

## Disclosure

AI Use Statement: The authors declare that no generative AI or AI‐assisted technologies were used in the preparation of this manuscript.

## Ethics Statement

The NHANES protocol was approved by the National Center for Health Statistics Research Ethics Review Board, and written informed consent was obtained from all participants. The present study was a secondary analysis of publicly available de‐identified data and was therefore exempt from additional institutional review board approval.

## Conflicts of Interest

The authors declare no conflicts of interest.

## Supporting information


**Figure S1:** Class distribution in the training set before and after applying the Synthetic Minority Over‐sampling Technique (SMOTE) for MetS–KSD comorbidity prediction.

**Figure S2:** Feature preprocessing and selection for MetS–KSD comorbidity modeling. (A) VIF‐based assessment of multicollinearity among candidate features. (B) Boruta feature selection results showing confirmed retained features. (C) Iterative Z‐score trajectories across Boruta iterations.

**Figure S3:** Mean absolute SHAP values of the top 15 features under two modeling strategies. (A) Random Forest model incorporating demographic variables and dietary micronutrients. (B) XGBoost model using dietary micronutrients alone.

**Figure S4:** Case‐level SHAP explanation for the Random Forest model incorporating demographic and dietary variables. (A) Force plot. (B) Waterfall plot.

**Figure S5:** Case‐level SHAP explanation for the XGBoost model using dietary micronutrients alone. (A) Force plot. (B) Waterfall plot.

**Figure S6:** SHAP dependence plots for key dietary micronutrients under two modeling strategies. (A) Random Forest model incorporating demographic and dietary variables. (B) XGBoost model using dietary micronutrients alone.

**Figure S7:** LIME‐based local interpretation for the Random Forest model incorporating demographic and dietary variables. (A) Local prediction probability for the illustrative case. (B) Local feature contribution plot.

**Figure S8:** LIME‐based local interpretation for the XGBoost model using dietary micronutrients alone. (A) Local prediction probability for the illustrative case. (B) Local feature contribution plot.

**Figure S9:** ROC and PR curves of the reduced‐feature models constructed using the top 15 features ranked by the SHAP algorithm under two modeling strategies. (A) ROC curves for models incorporating demographic characteristics and dietary micronutrients; (B) PR curves for models incorporating demographic characteristics and dietary micronutrients; (C) ROC curves for models based on dietary micronutrients alone; (D) PR curves for models based on dietary micronutrients alone.

**Figure S10:** Feature selection using LASSO regression. (A) Cross‐validation curve for the LASSO model incorporating demographic characteristics and dietary micronutrients; (B) coefficient path for the LASSO model incorporating demographic characteristics and dietary micronutrients; (C) cross‐validation curve for the LASSO model based on dietary micronutrients alone; (D) coefficient path for the LASSO model based on dietary micronutrients alone.

**Figure S11:** Model performance and SHAP‐based interpretability analyses following LASSO feature selection. (A) ROC curves for models incorporating demographic characteristics and dietary micronutrients; (B) PR curves for models incorporating demographic characteristics and dietary micronutrients; (C) SHAP summary plot for the LASSO‐selected model incorporating demographic characteristics and dietary micronutrients; (D) ROC curves for models based on dietary micronutrients alone; (E) PR curves for models based on dietary micronutrients alone; (F) SHAP summary plot for the LASSO‐selected model based on dietary micronutrients alone.

**Figure S12:** Comparison of model performance before and after SMOTE processing. (A) ROC curves for models incorporating demographic characteristics and dietary micronutrients after SMOTE processing; (B) ROC curves for models incorporating demographic characteristics and dietary micronutrients without SMOTE processing; (C) PR curves for models incorporating demographic characteristics and dietary micronutrients after SMOTE processing; (D) PR curves for models incorporating demographic characteristics and dietary micronutrients without SMOTE processing; (E) ROC curves for models based on dietary micronutrients alone after SMOTE processing; (F) ROC curves for models based on dietary micronutrients alone without SMOTE processing; (G) PR curves for models based on dietary micronutrients alone after SMOTE processing; (H) PR curves for models based on dietary micronutrients alone without SMOTE processing.

**Table S1:** Comparative performance of six SMOTE‐balanced machine‐learning models for predicting MetS–KSD comorbidity using demographic variables and dietary micronutrients.

**Table S2:** Comparative performance of six SMOTE‐balanced machine‐learning models for predicting MetS–KSD comorbidity using dietary micronutrients alone.

**Table S3:** Performance of reduced‐feature machine‐learning models constructed using the top 15 SHAP‐ranked variables under two modeling strategies.

**Table S4:** Performance of LASSO‐based reduced‐feature machine‐learning models under two modeling strategies.

**Table S5:** Performance of machine‐learning models trained without SMOTE processing under two modeling strategies.

## Data Availability

The data that support the findings of this study are available in National Health and Nutrition Examination Survey (NHANES) at https://www.cdc.gov/nchs/nhanes/. These data were derived from the following resources available in the public domain: Centers for Disease Control and Prevention (CDC), https://www.cdc.gov/nchs/nhanes/.
