# Supplementary figures and images for: Interpretable Machine Learning for Predicting Metabolic Syndrome–Kidney Stone Disease Comorbidity: The Role of Dietary Micronutrients
Source: Food Sci Nutr. 2026 Jun 10;14(6):e72019. doi: 10.1002/fsn3.72019 (PMC13253607; doi:10.1002/fsn3.72019)

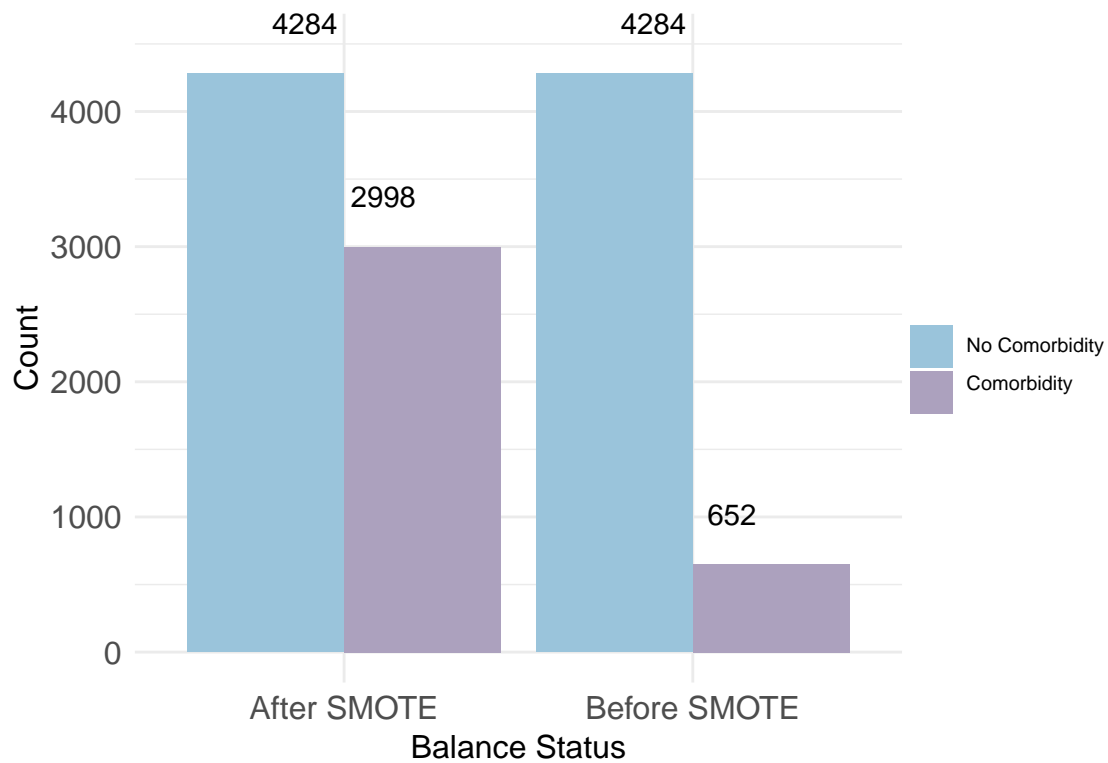

Supplement: Supplementary file 1 — Figure S1: Class distribution in the training set before and after applying the Synthetic Minority Over‐sampling Technique (SMOTE) for MetS–KSD comorbidity prediction. [file FSN3-14-e72019-s004.pdf]

A

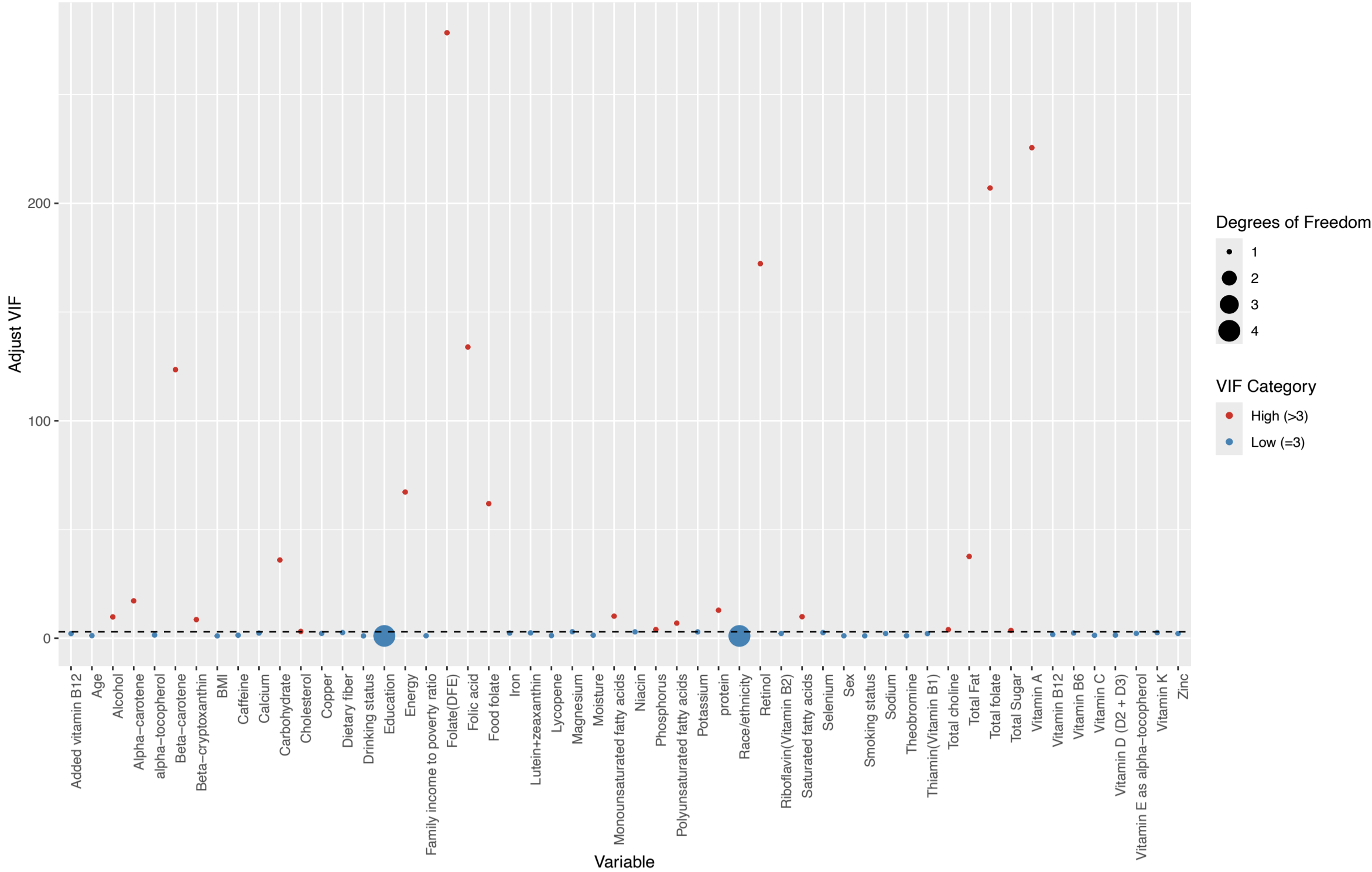

B

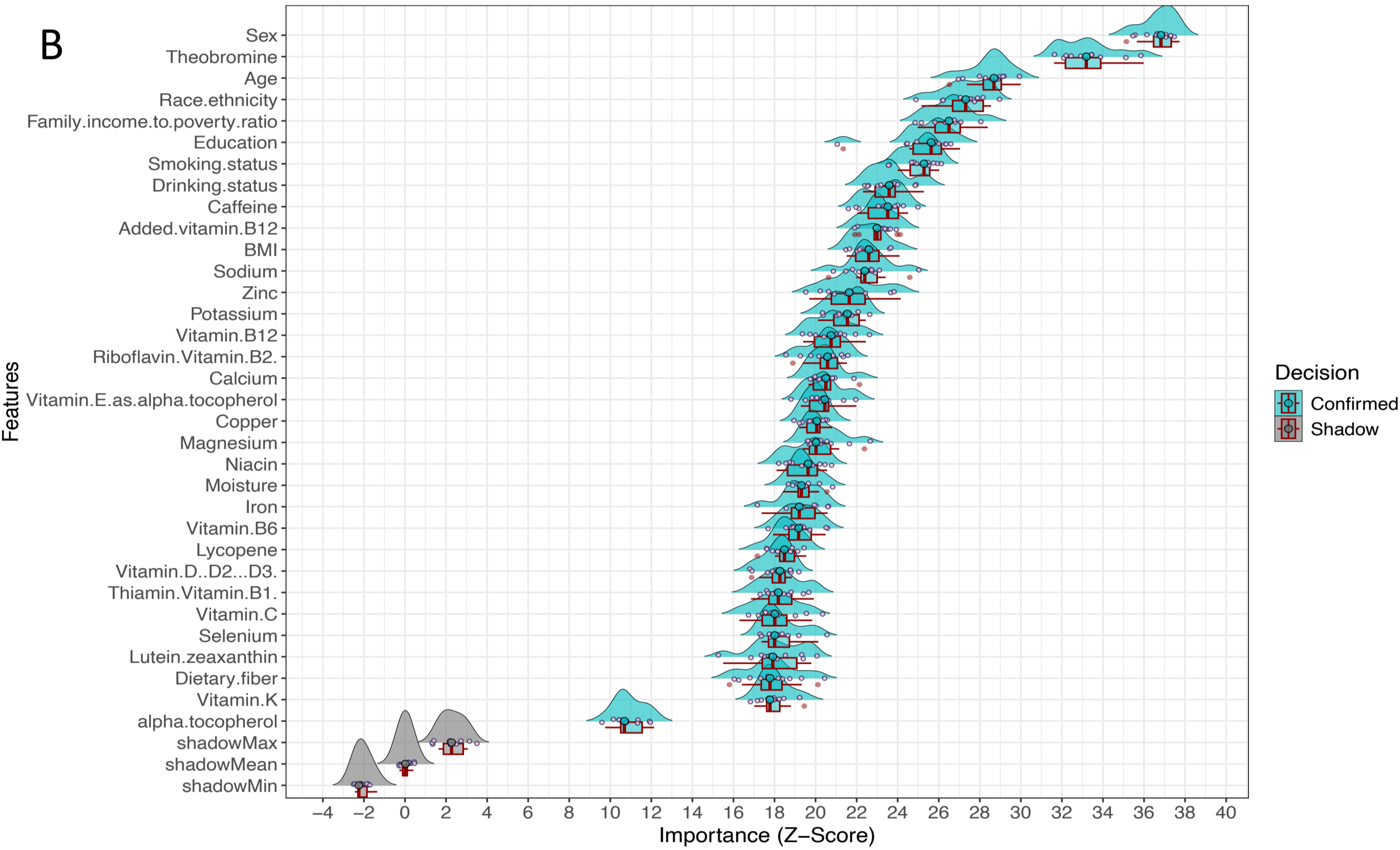

C

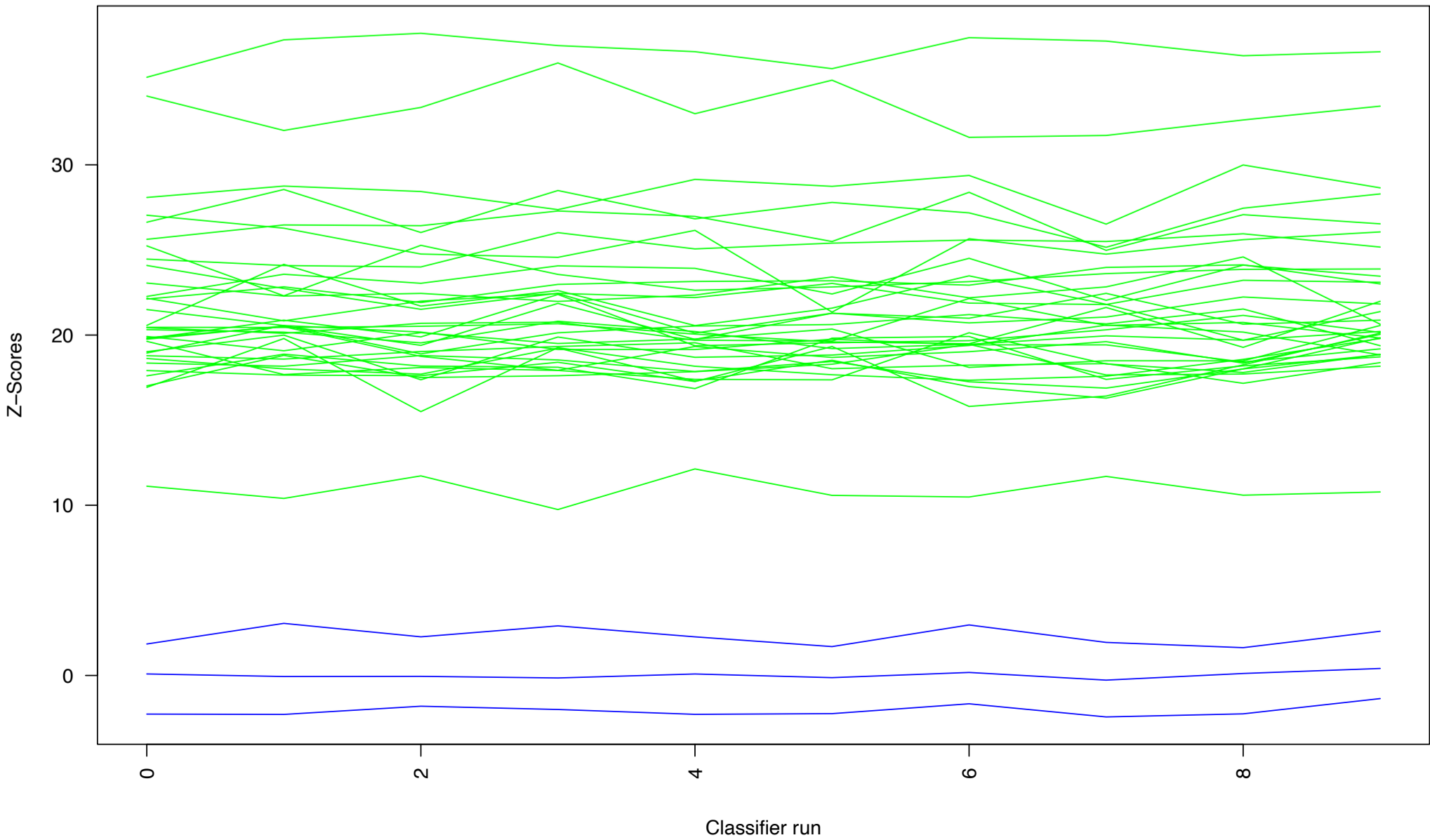

Supplement: Supplementary file 2 — Figure S2: Feature preprocessing and selection for MetS–KSD comorbidity modeling. (A) VIF‐based assessment of multicollinearity among candidate features. (B) Boruta feature selection results showing confirmed retained features. (C) Iterative Z‐score trajectories across Boruta iterations. [file FSN3-14-e72019-s010.pdf]

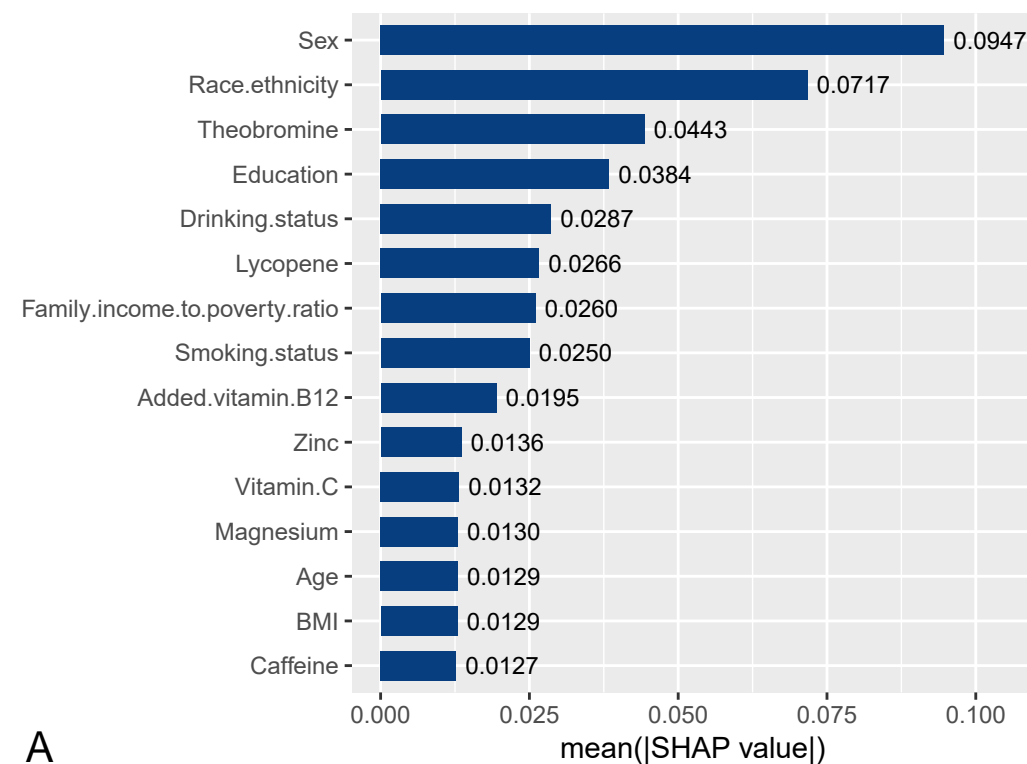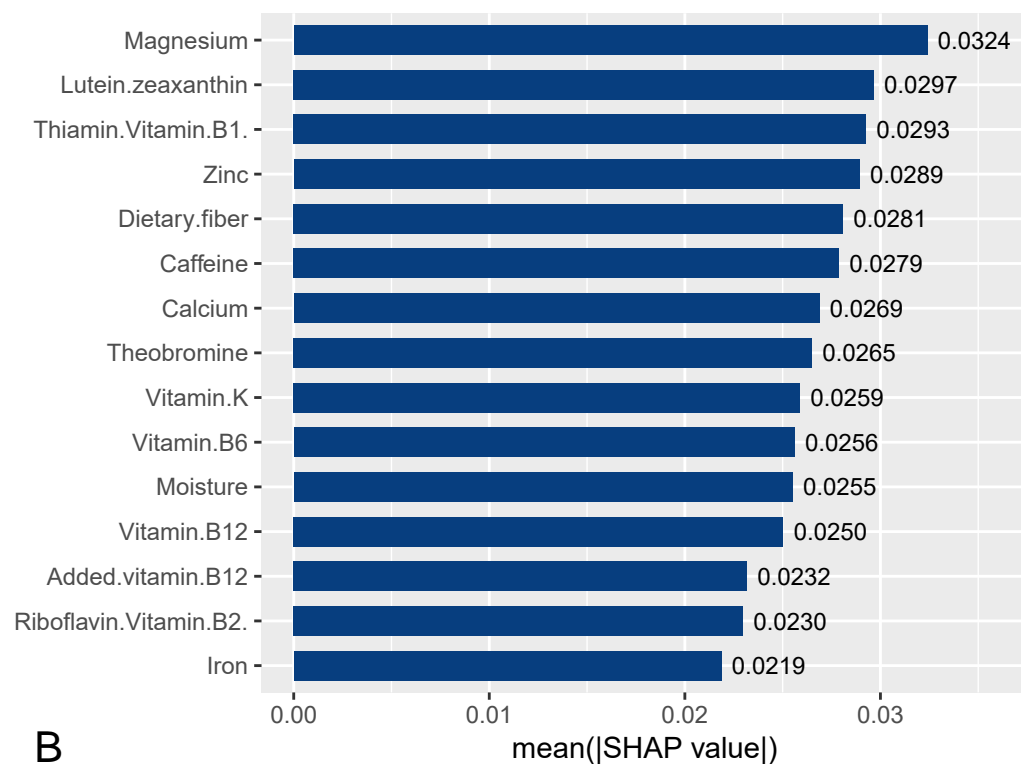

Supplement: Supplementary file 3 — Figure S3: Mean absolute SHAP values of the top 15 features under two modeling strategies. (A) Random Forest model incorporating demographic variables and dietary micronutrients. (B) XGBoost model using dietary micronutrients alone. [file FSN3-14-e72019-s016.pdf]

A

No comorbidities

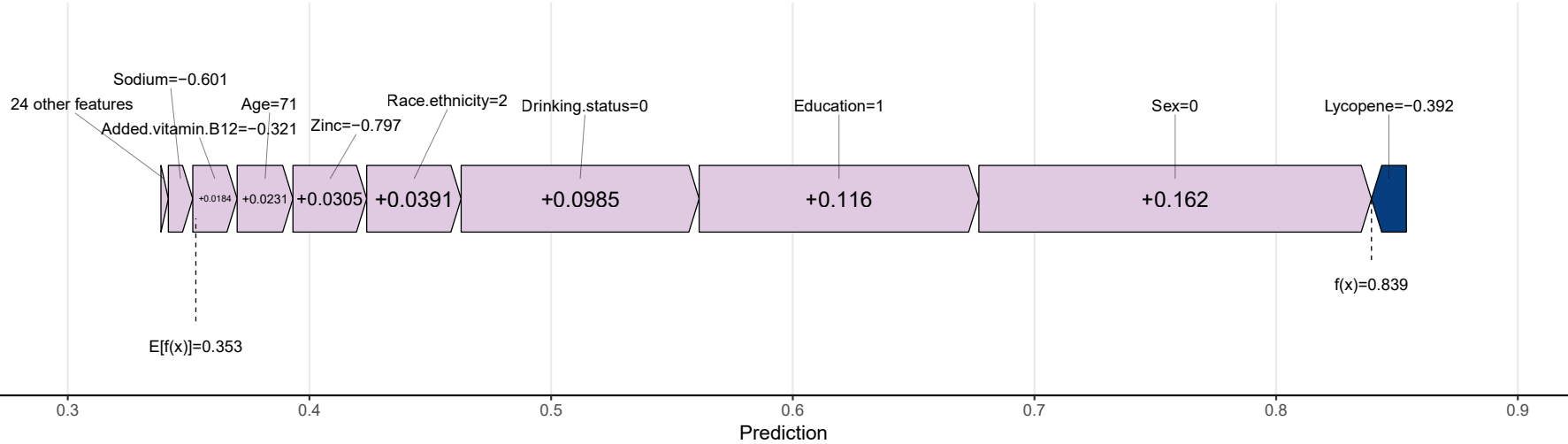

Comorbidities

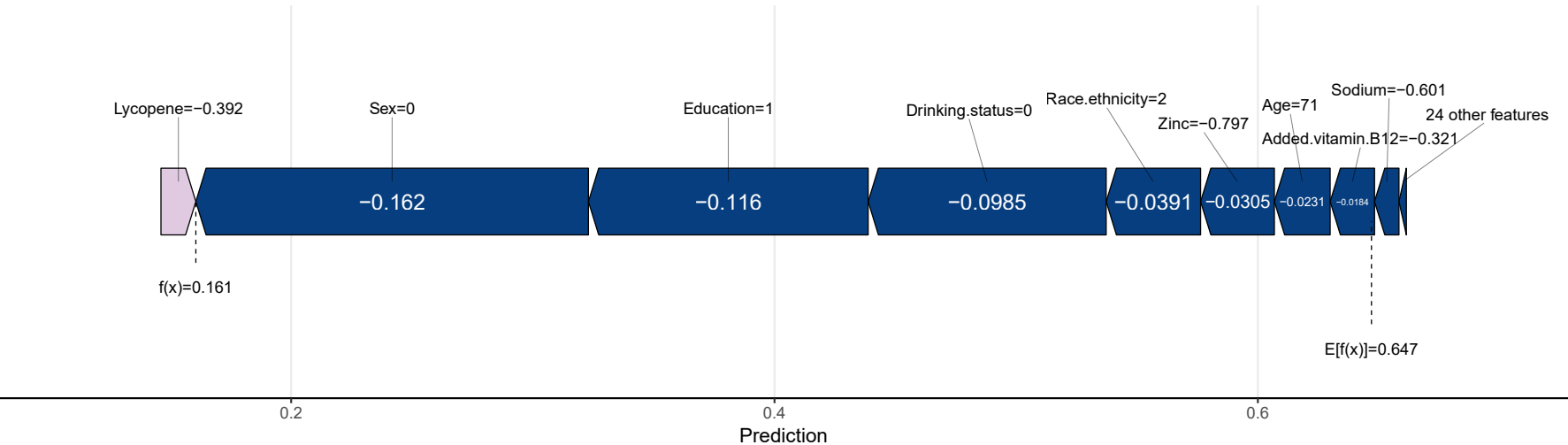

B

No comorbidities

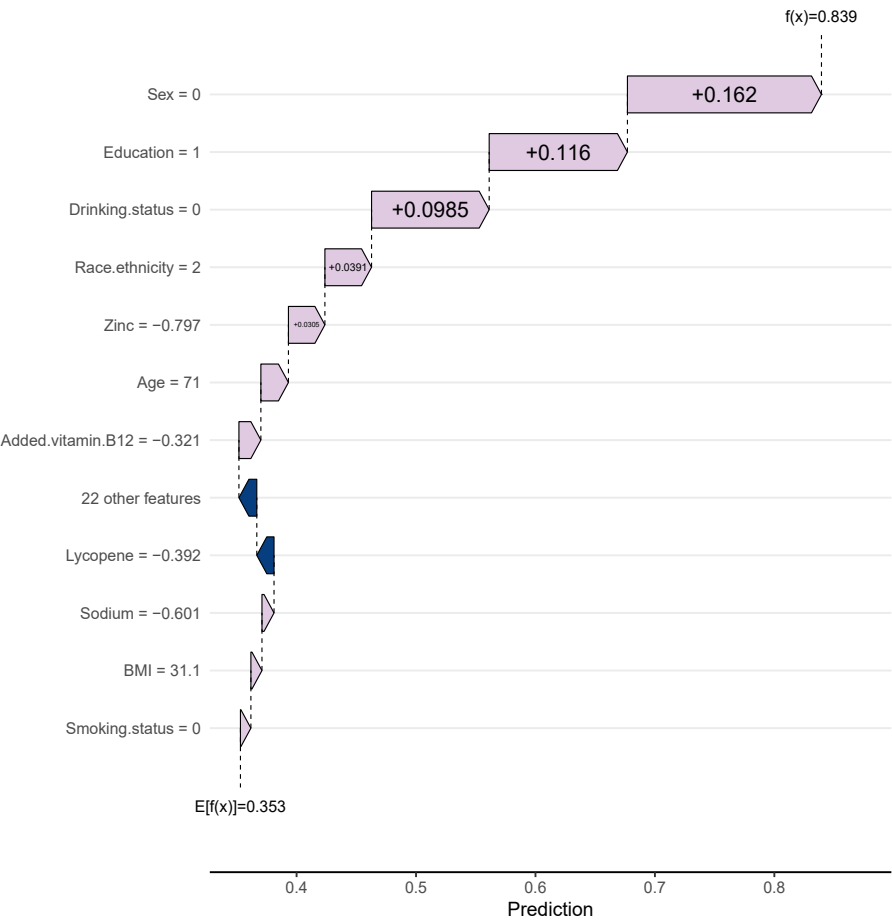

Comorbidities

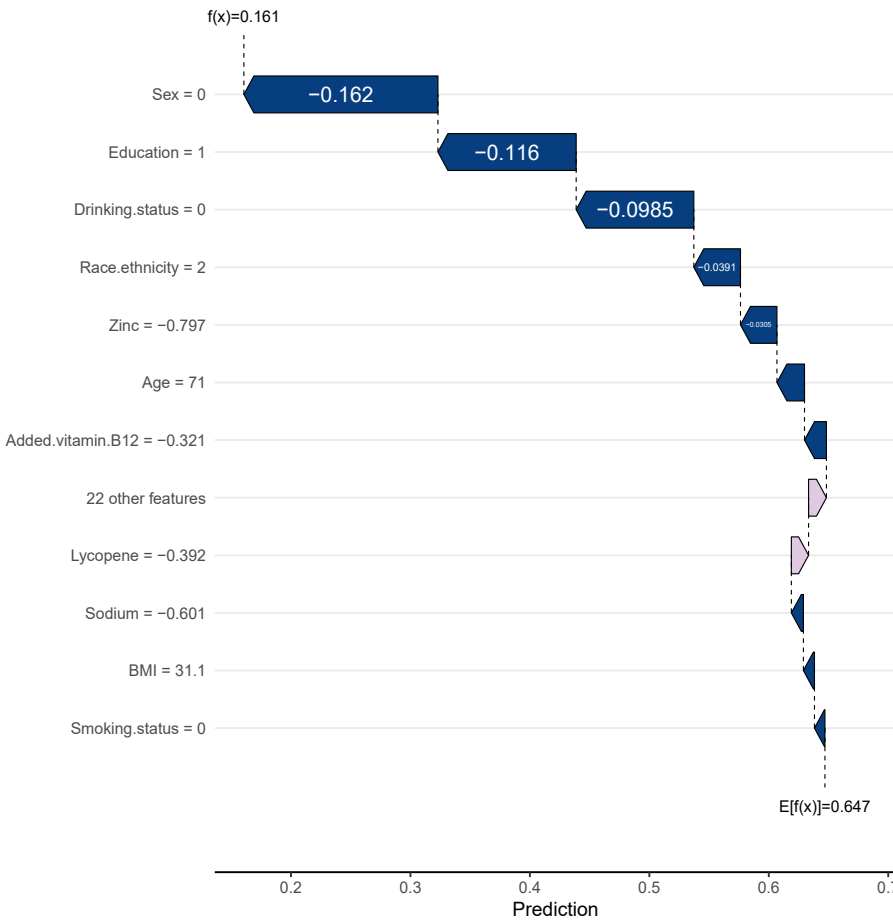

Supplement: Supplementary file 4 — Figure S4: Case‐level SHAP explanation for the Random Forest model incorporating demographic and dietary variables. (A) Force plot. (B) Waterfall plot. [file FSN3-14-e72019-s009.pdf]

A

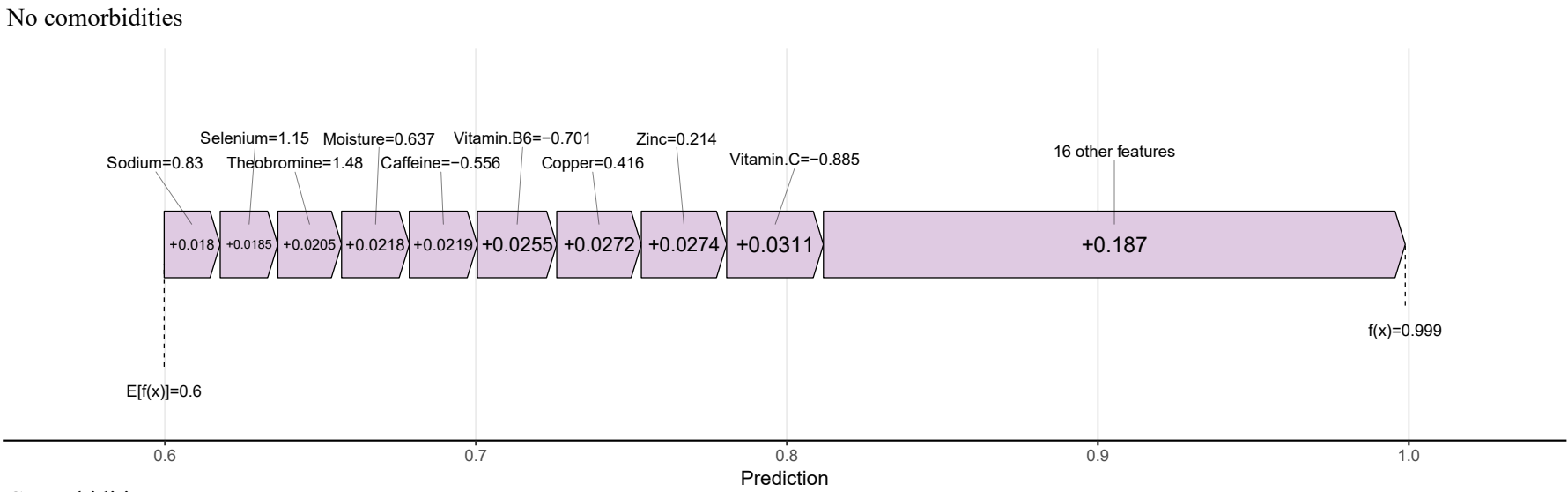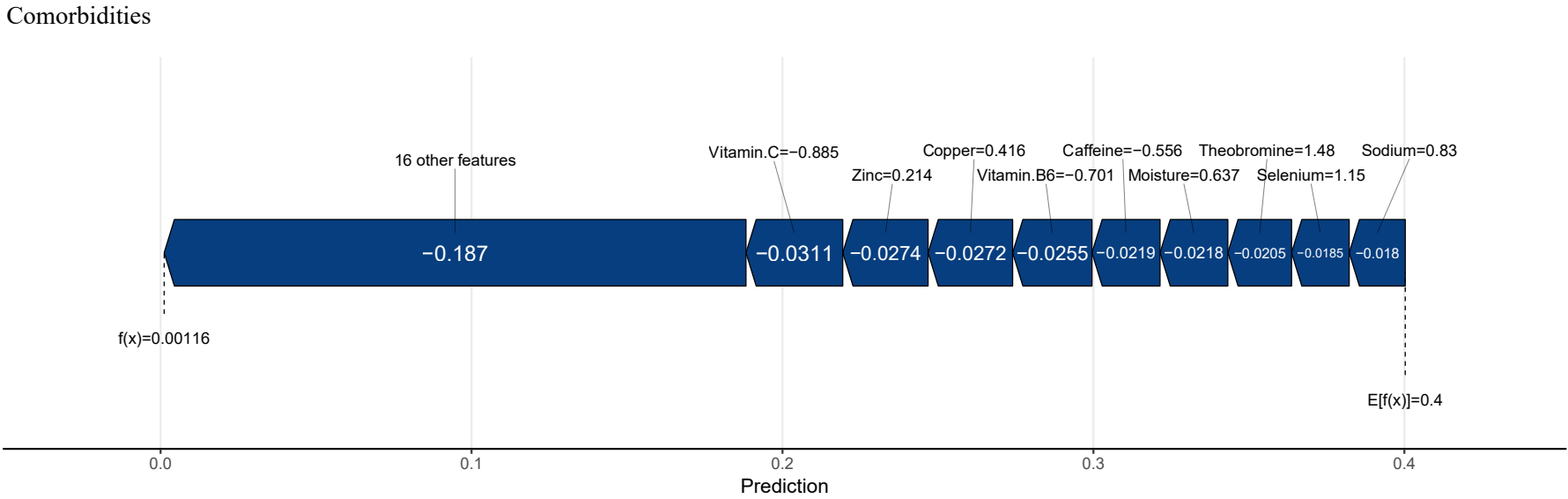

B

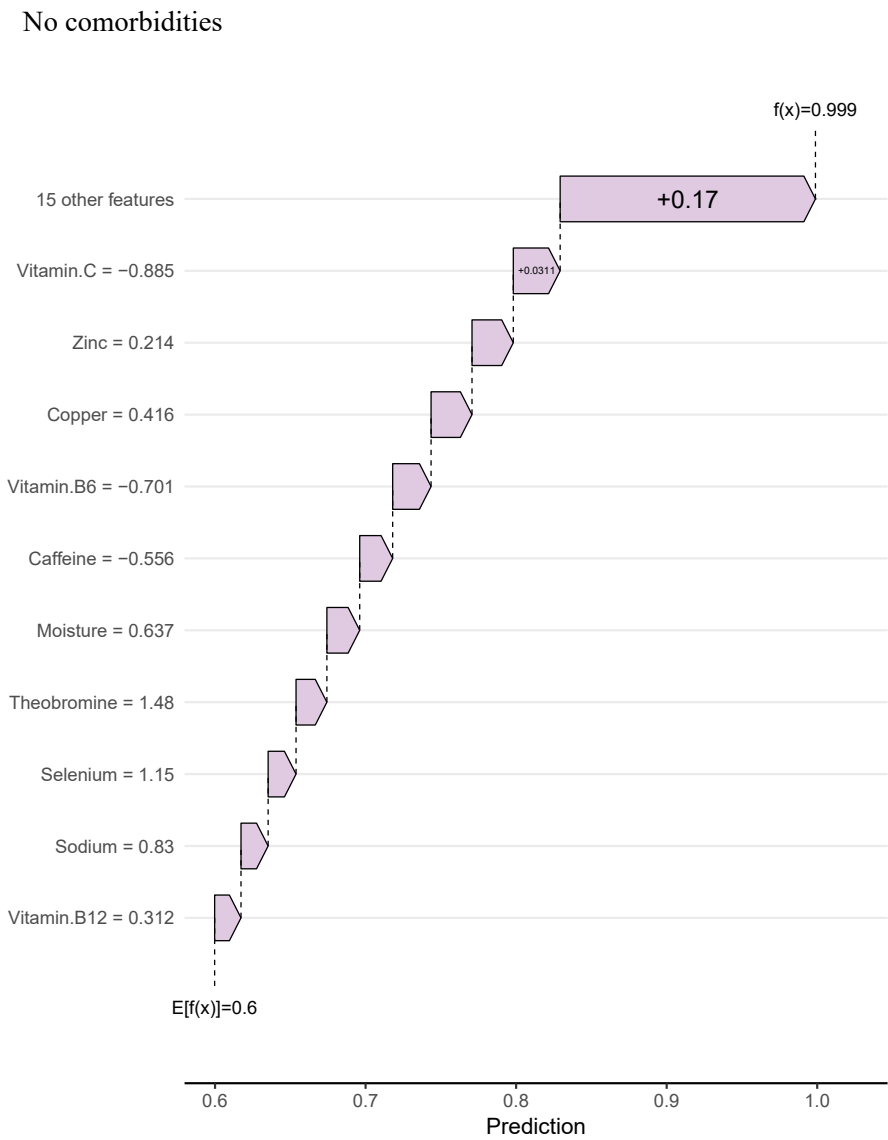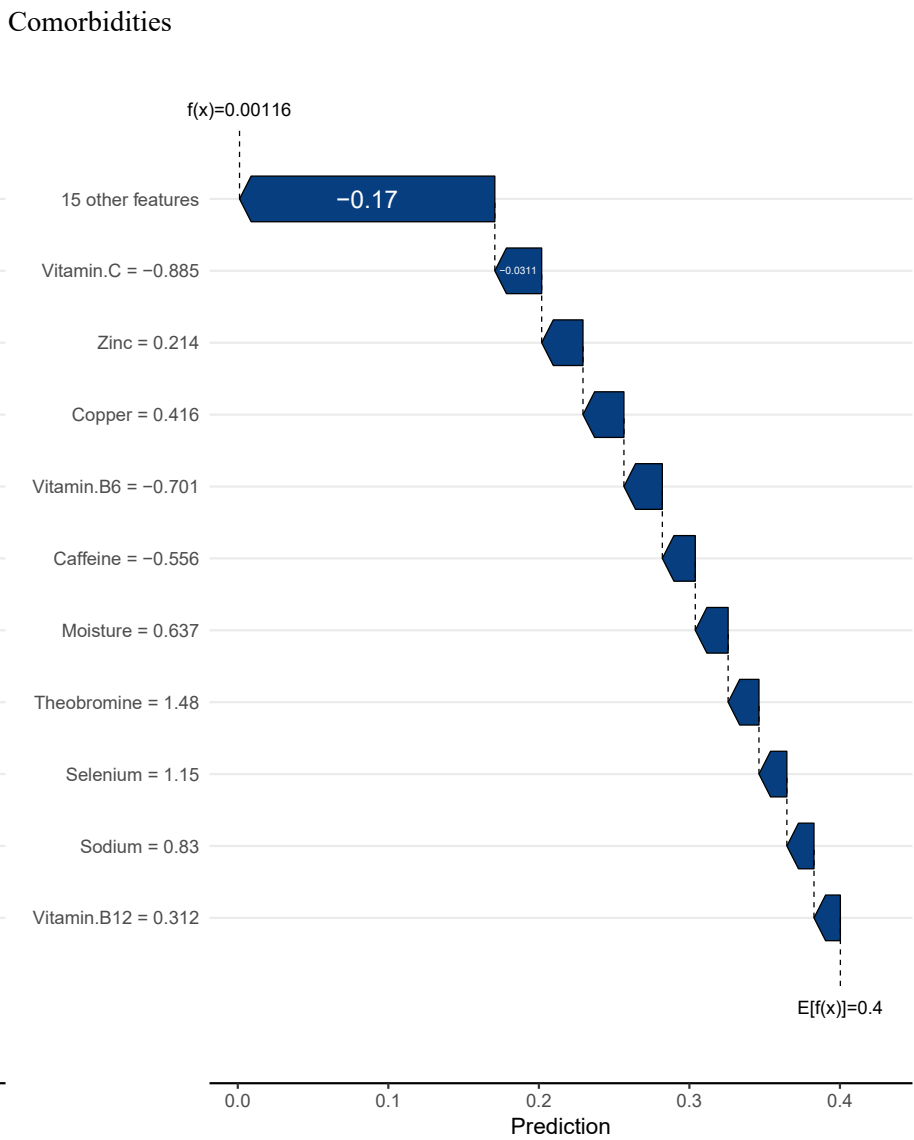

Supplement: Supplementary file 5 — Figure S5: Case‐level SHAP explanation for the XGBoost model using dietary micronutrients alone. (A) Force plot. (B) Waterfall plot. [file FSN3-14-e72019-s005.pdf]

A

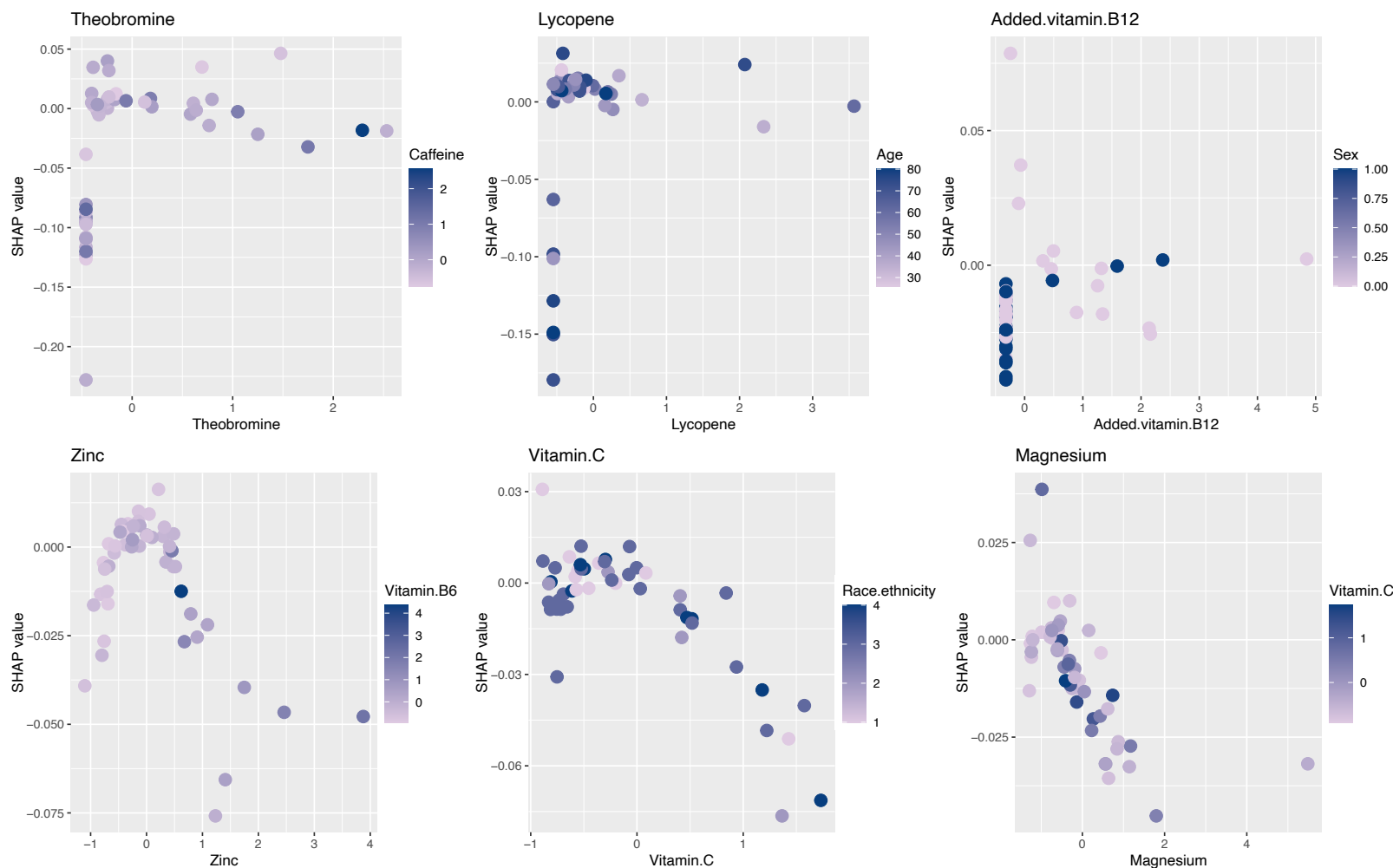

B

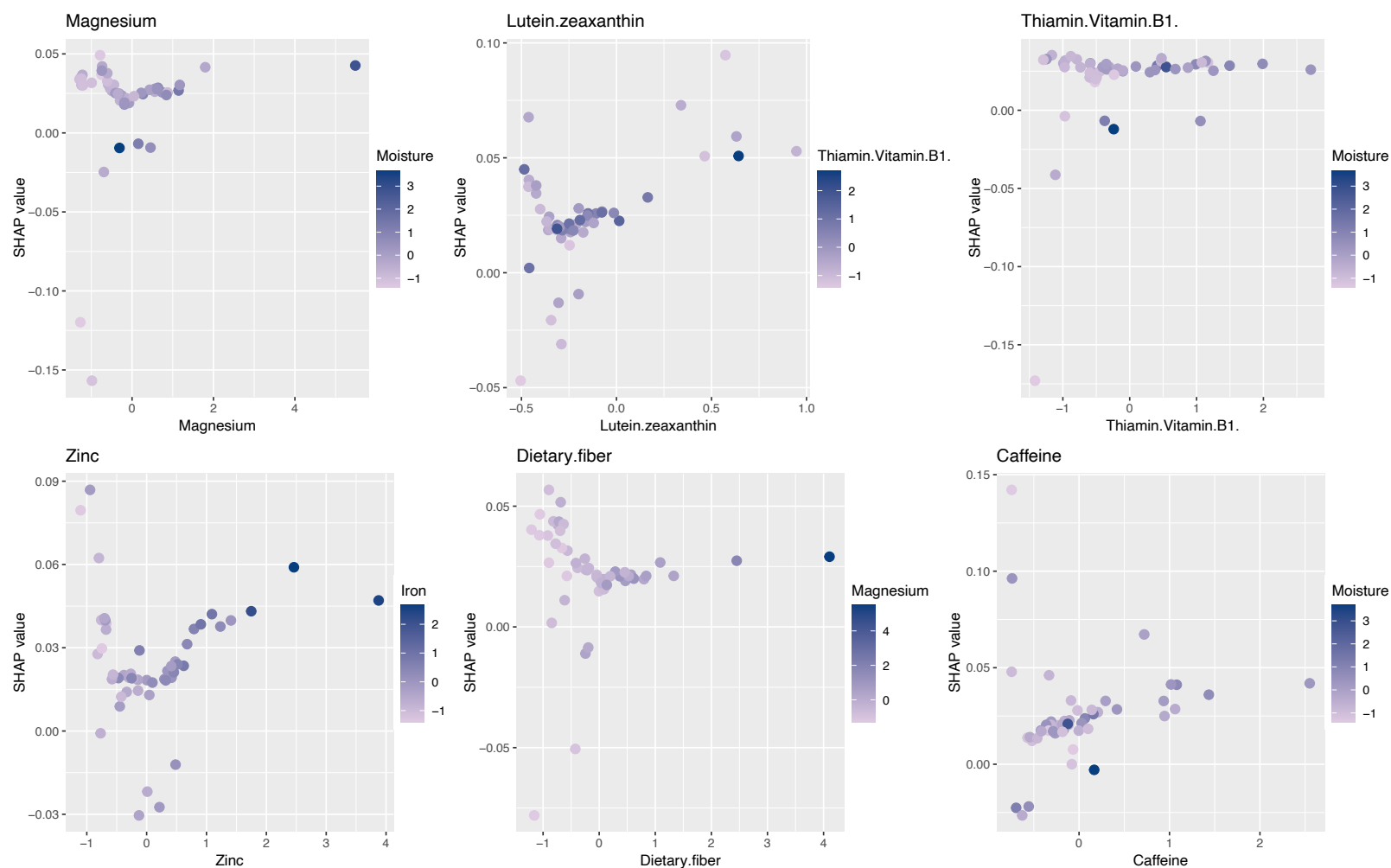

Supplement: Supplementary file 6 — Figure S6: SHAP dependence plots for key dietary micronutrients under two modeling strategies. (A) Random Forest model incorporating demographic and dietary variables. (B) XGBoost model using dietary micronutrients alone. [file FSN3-14-e72019-s007.pdf]

A

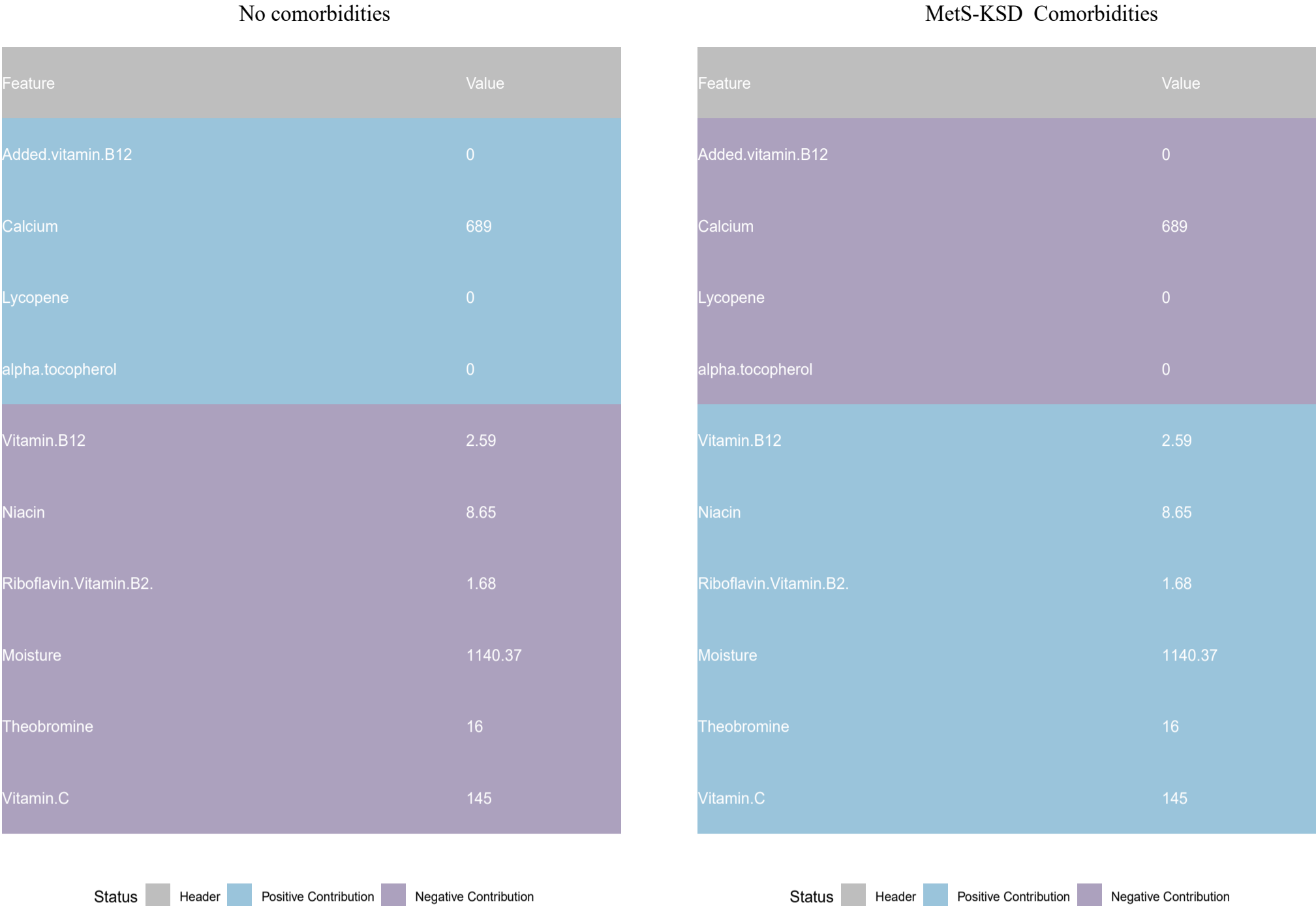

B

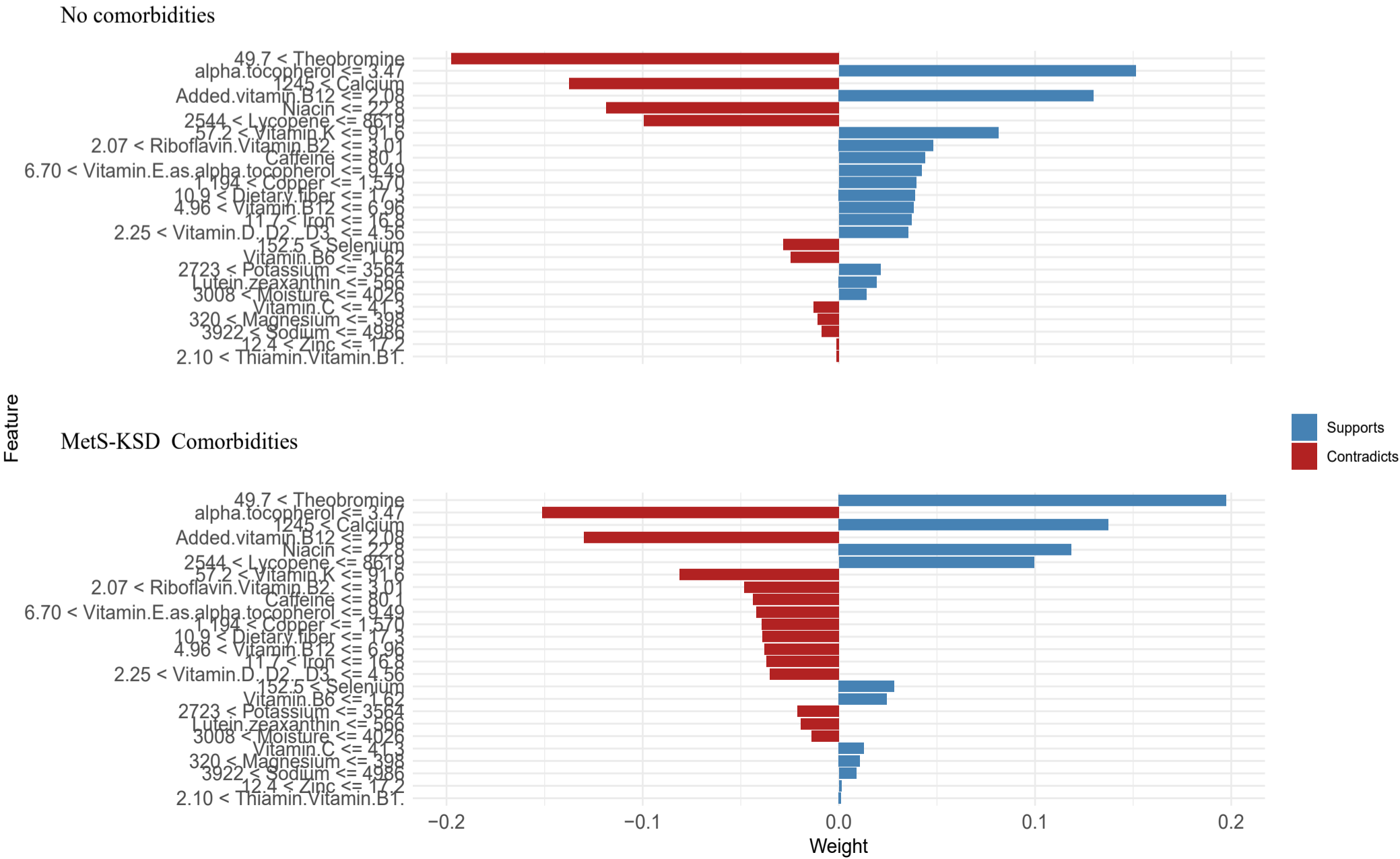

Supplement: Supplementary file 8 — Figure S8: LIME‐based local interpretation for the XGBoost model using dietary micronutrients alone. (A) Local prediction probability for the illustrative case. (B) Local feature contribution plot. [file FSN3-14-e72019-s011.pdf]

A

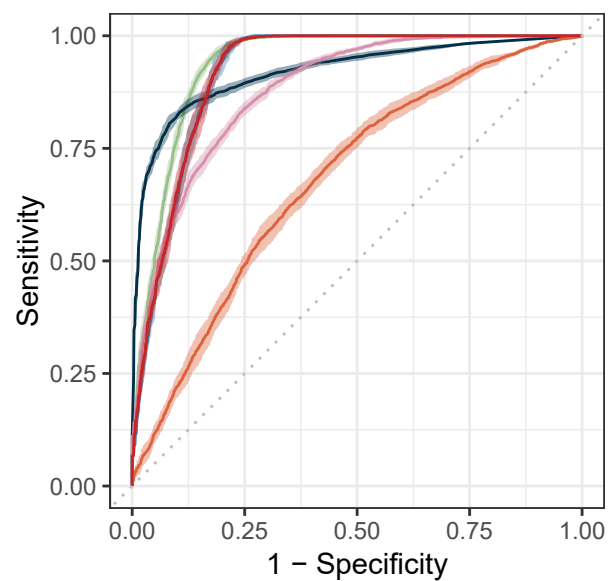

B

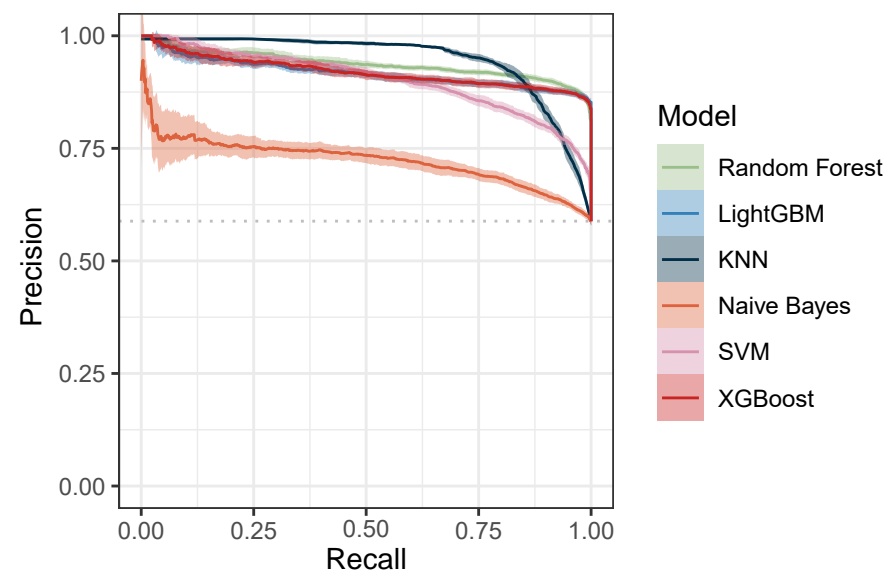

C

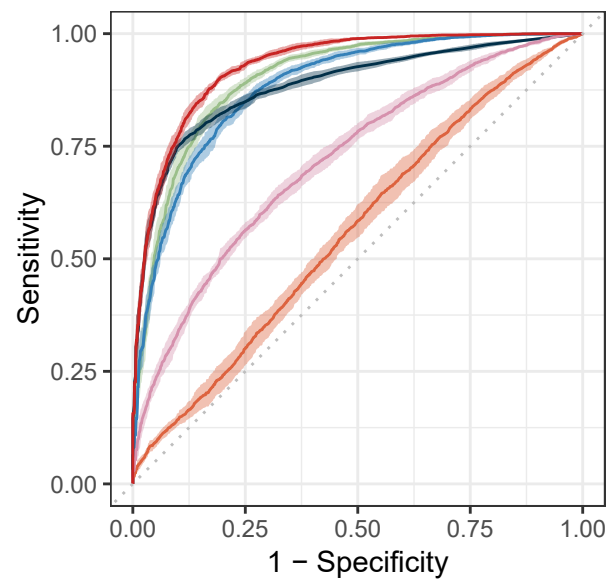

D

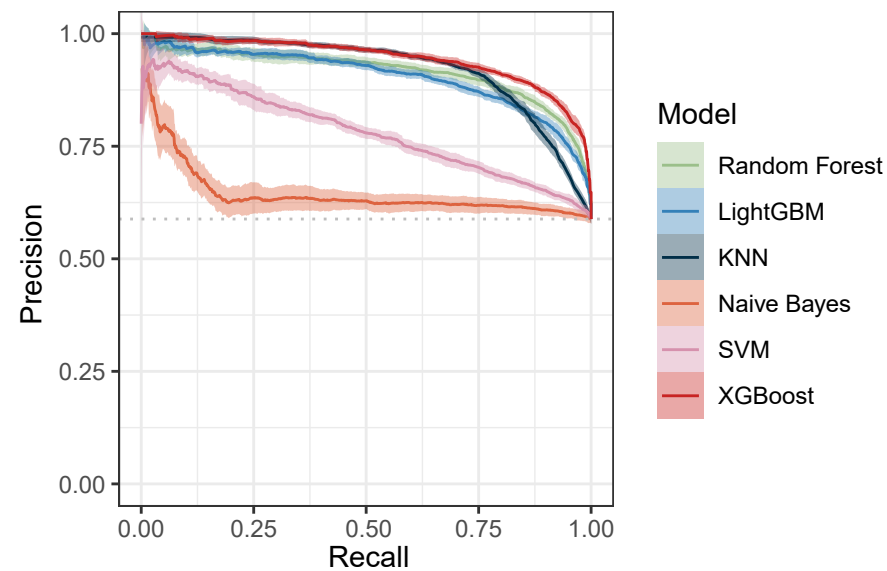

Supplement: Supplementary file 9 — Figure S9: ROC and PR curves of the reduced‐feature models constructed using the top 15 features ranked by the SHAP algorithm under two modeling strategies. (A) ROC curves for models incorporating demographic characteristics and dietary micronutrients; (B) PR curves for models incorporating demographic characteristics and dietary micronutrients; (C) ROC curves for models based on dietary micronutrients alone; (D) PR curves for models based on dietary micronutrients alone. [file FSN3-14-e72019-s002.pdf]

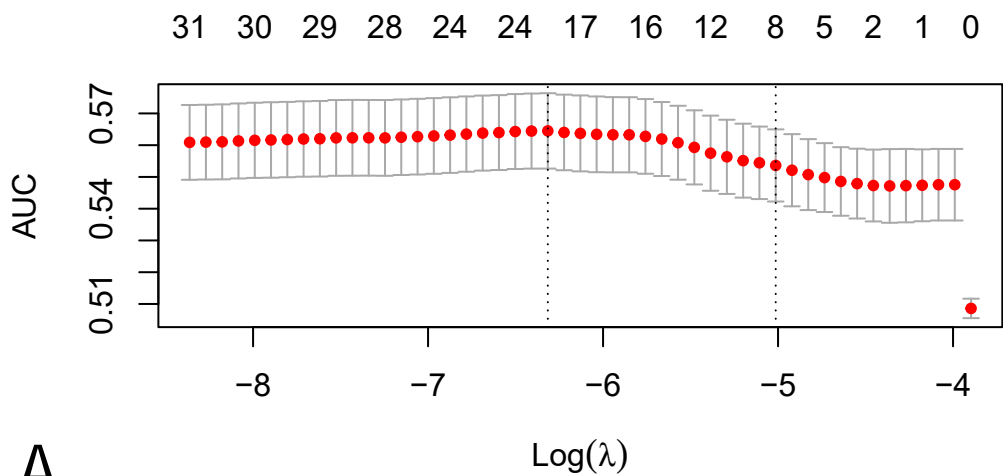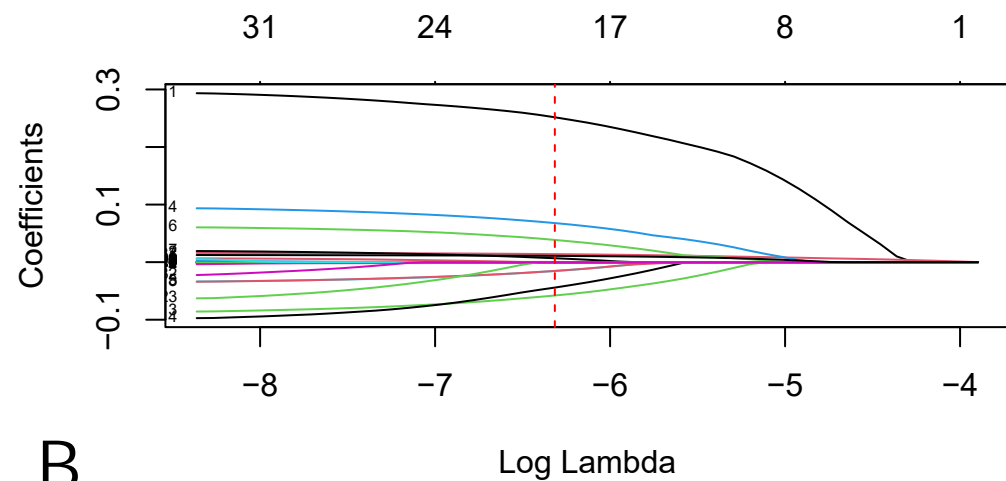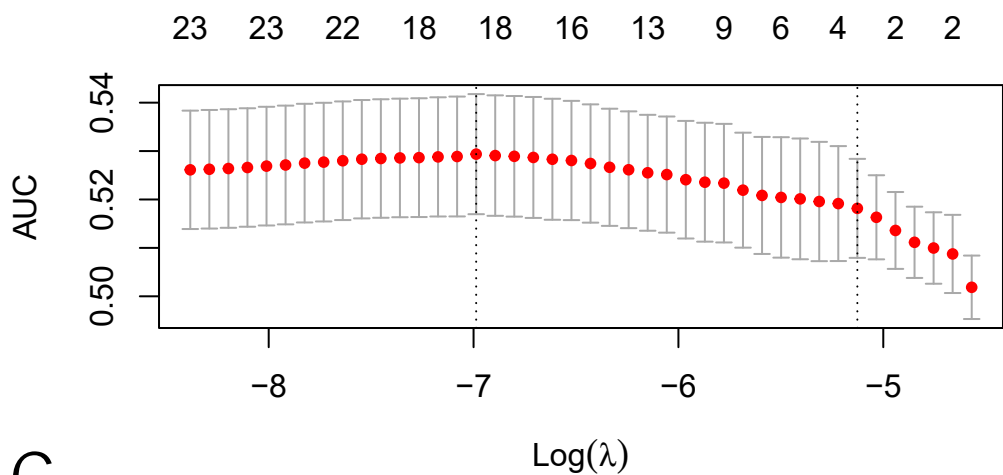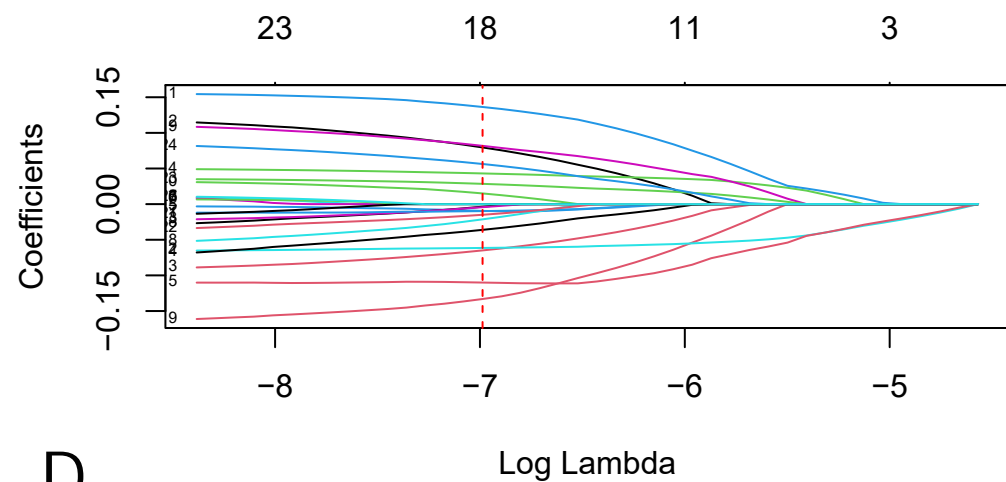

Supplement: Supplementary file 10 — Figure S10: Feature selection using LASSO regression. (A) Cross‐validation curve for the LASSO model incorporating demographic characteristics and dietary micronutrients; (B) coefficient path for the LASSO model incorporating demographic characteristics and dietary micronutrients; (C) cross‐validation curve for the LASSO model based on dietary micronutrients alone; (D) coefficient path for the LASSO model based on dietary micronutrients alone. [file FSN3-14-e72019-s013.pdf]

A

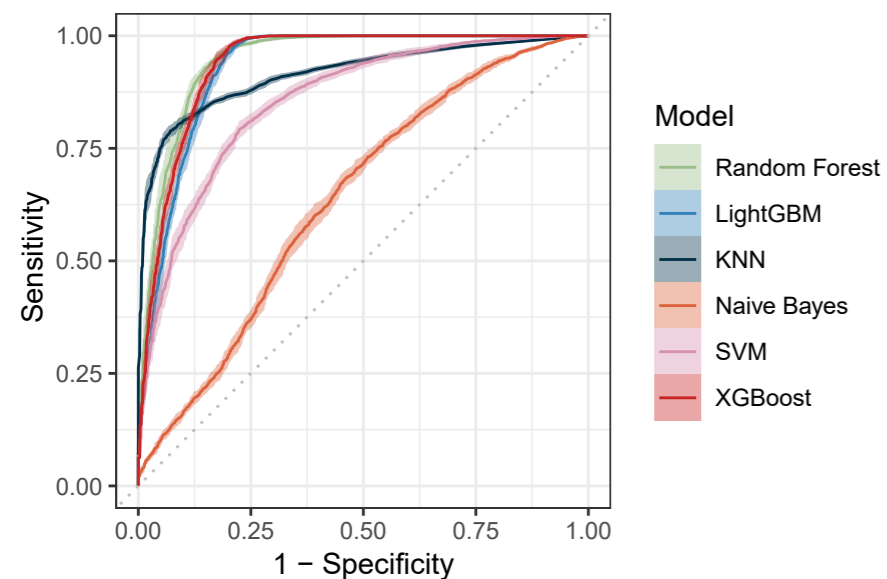

B

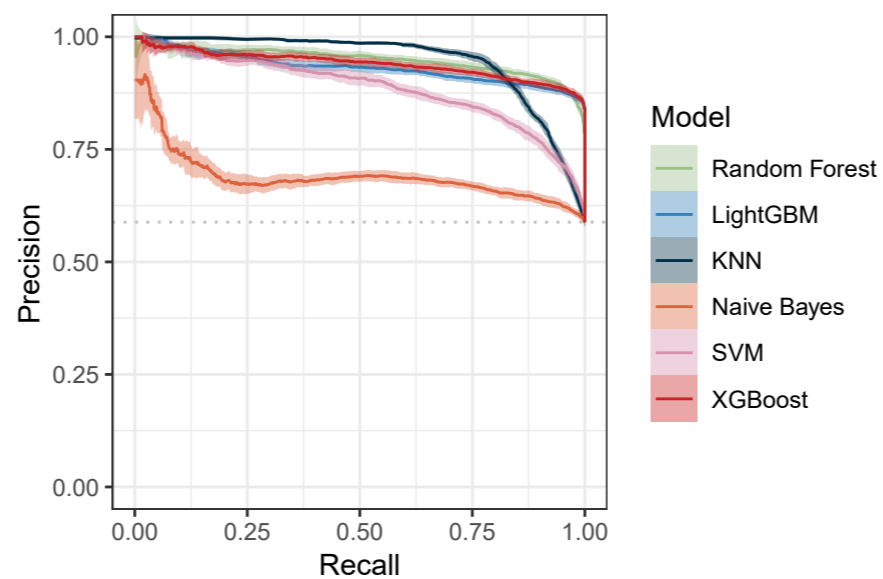

C

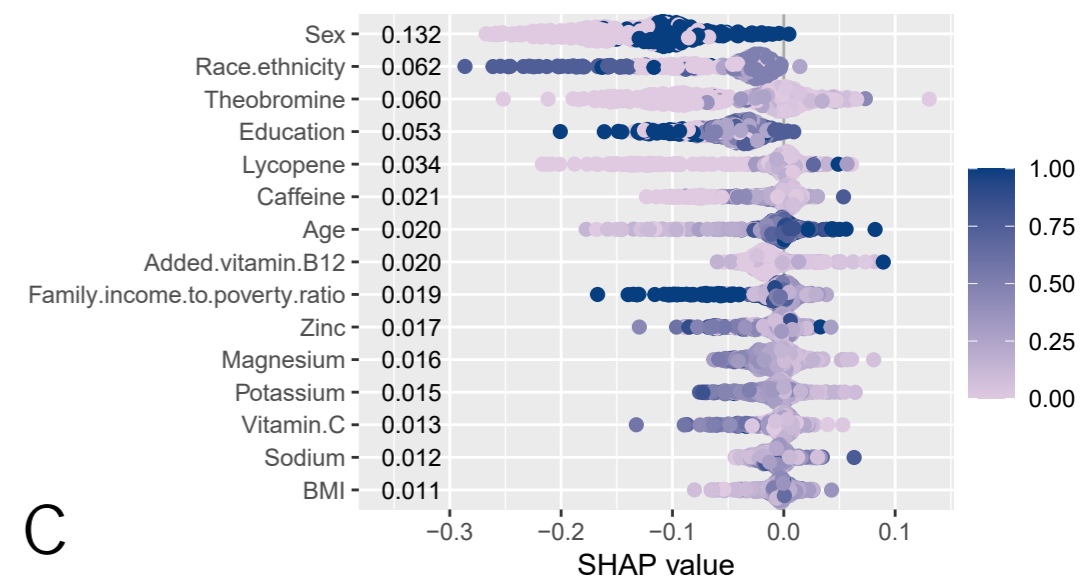

D

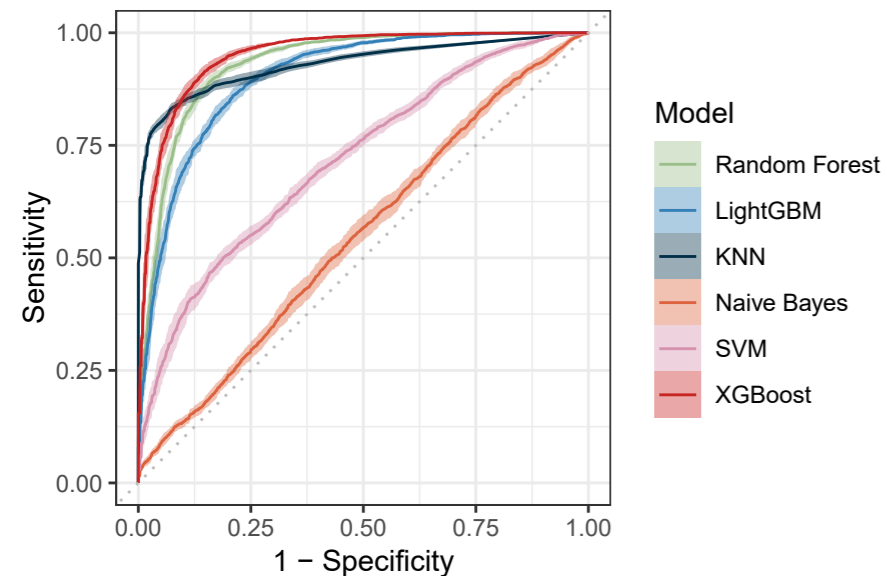

E

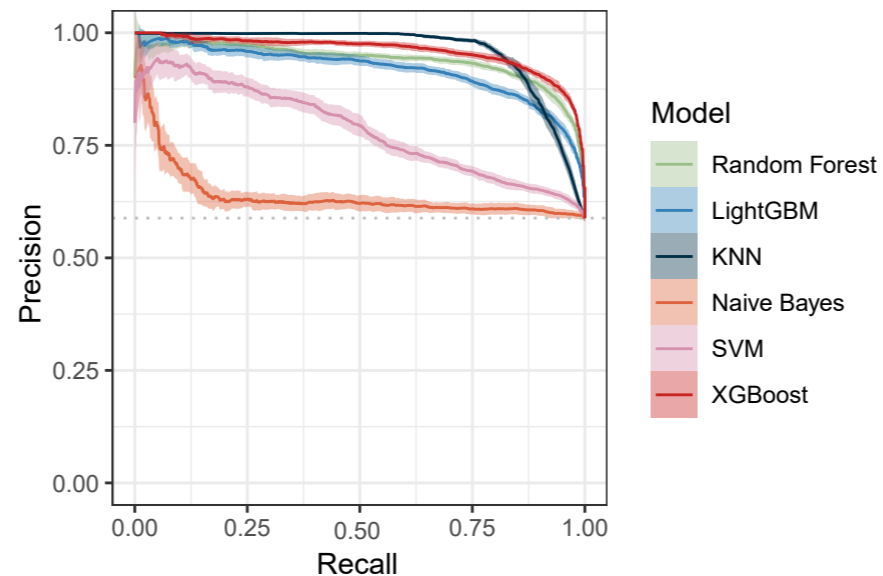

F

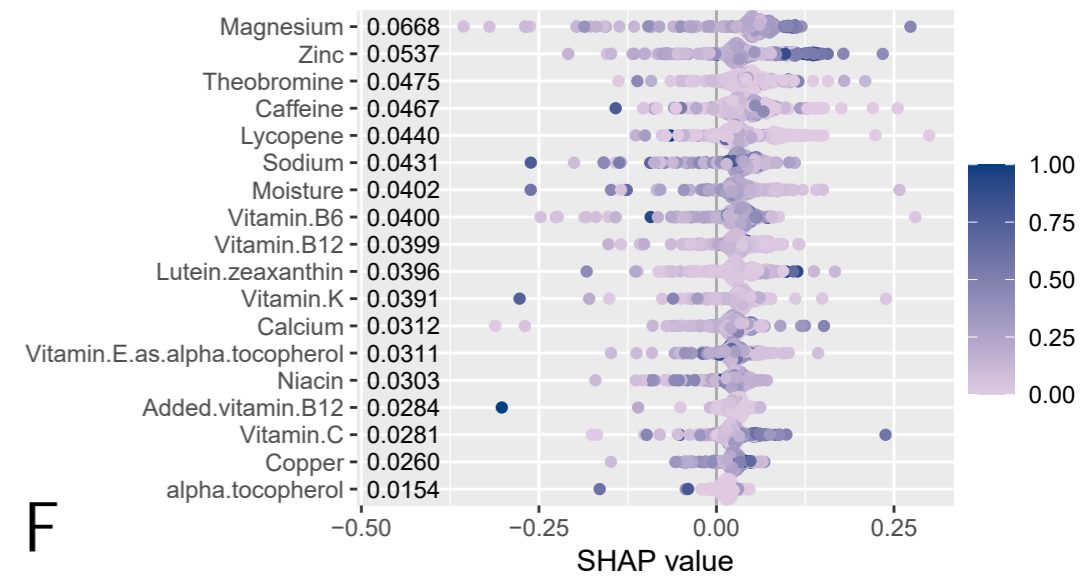

Supplement: Supplementary file 11 — Figure S11: Model performance and SHAP‐based interpretability analyses following LASSO feature selection. (A) ROC curves for models incorporating demographic characteristics and dietary micronutrients; (B) PR curves for models incorporating demographic characteristics and dietary micronutrients; (C) SHAP summary plot for the LASSO‐selected model incorporating demographic characteristics and dietary micronutrients; (D) ROC curves for models based on dietary micronutrients alone; (E) PR curves for models based on dietary micronutrients alone; (F) SHAP summary plot for the LASSO‐selected model based on dietary micronutrients alone. [file FSN3-14-e72019-s006.pdf]

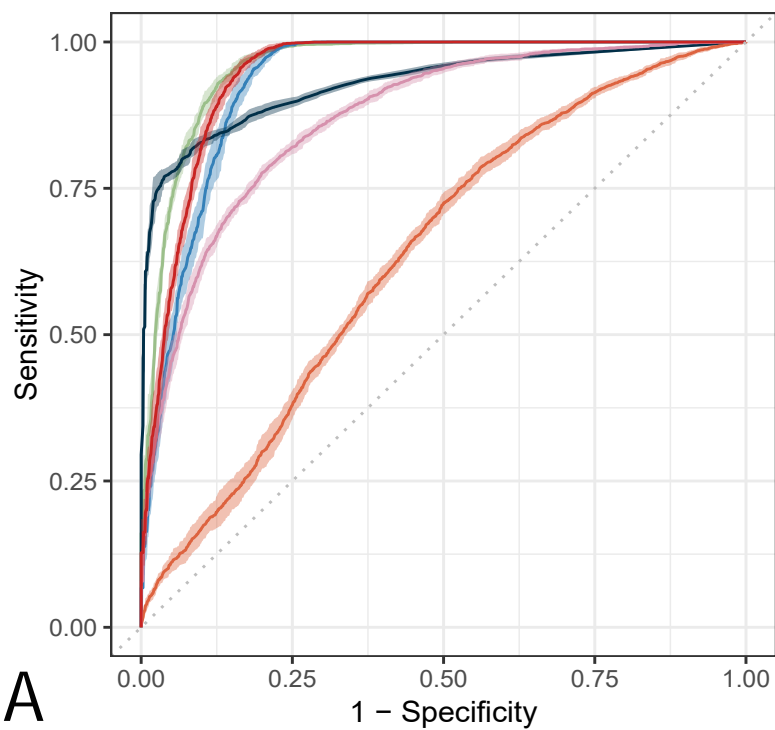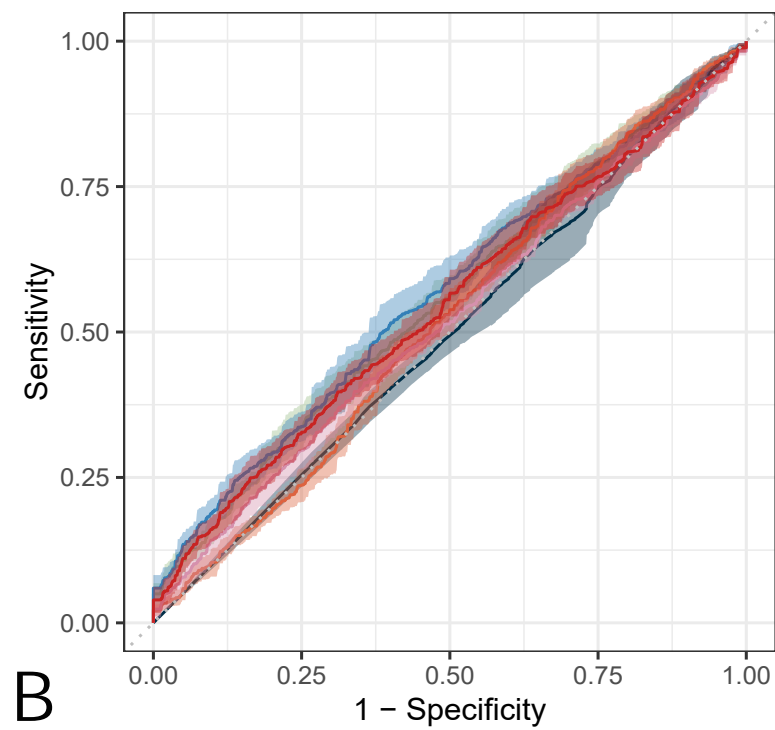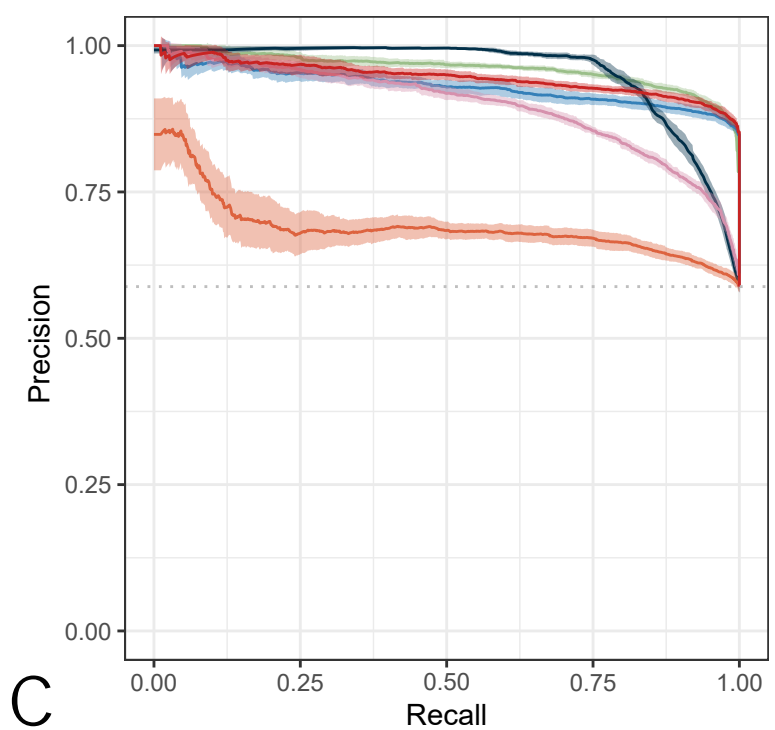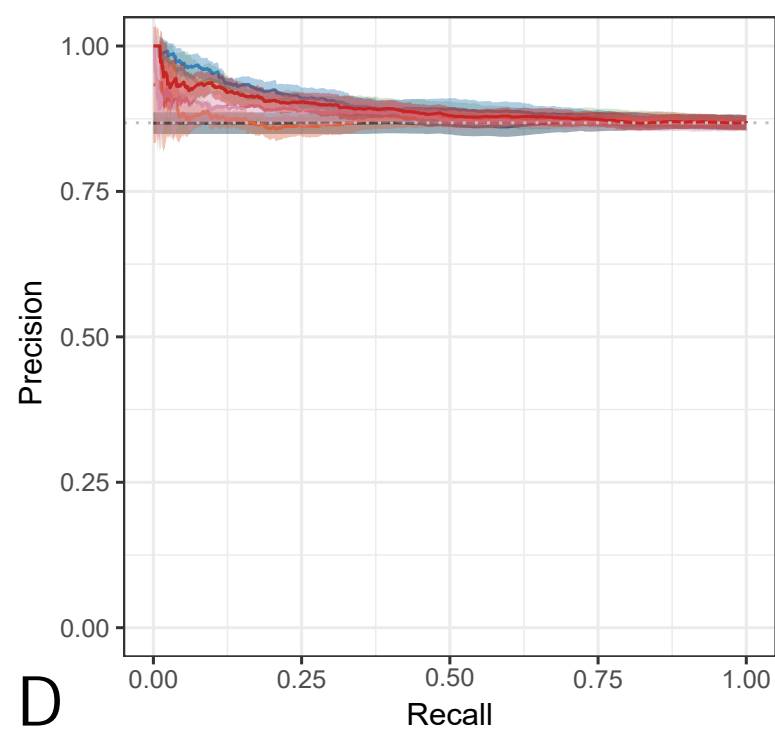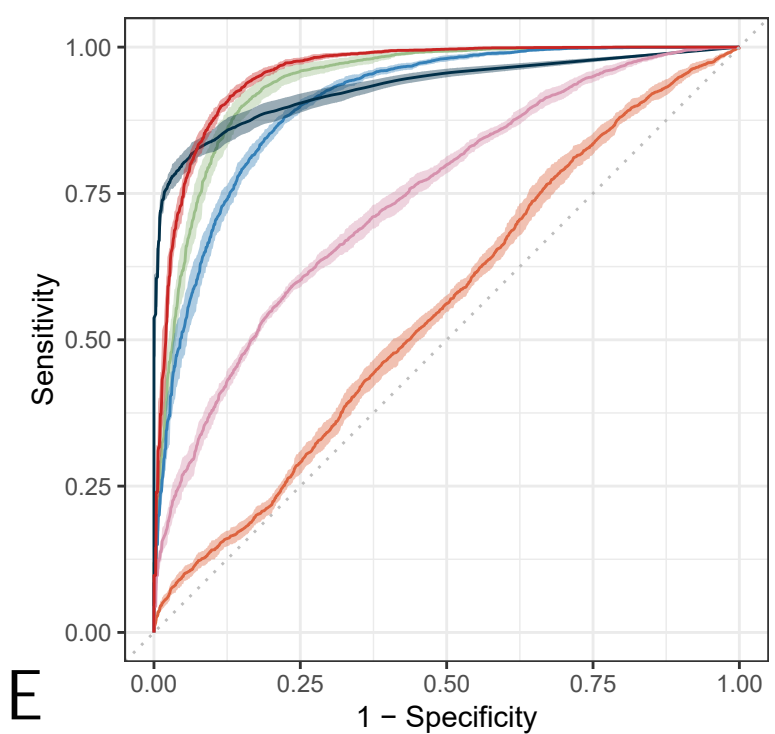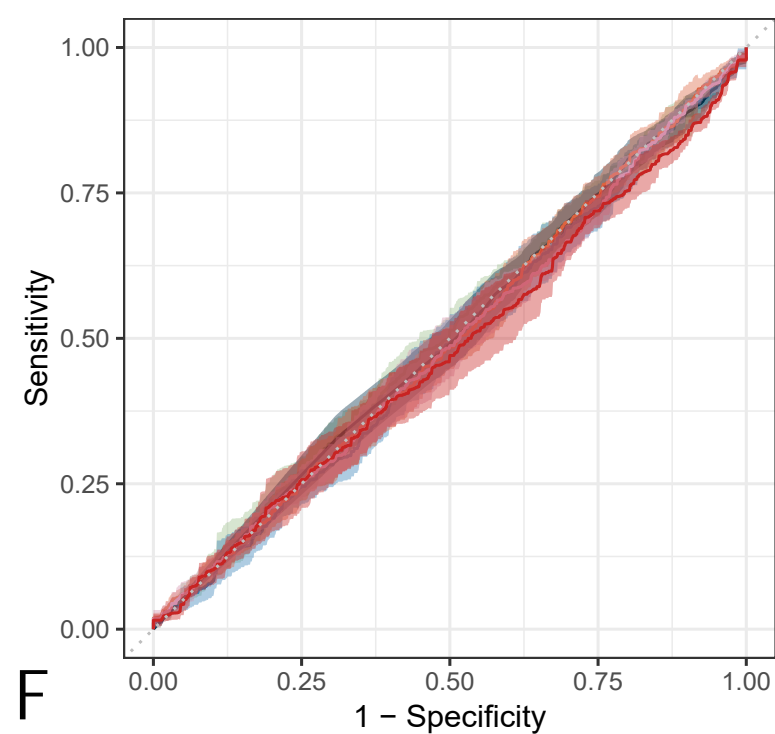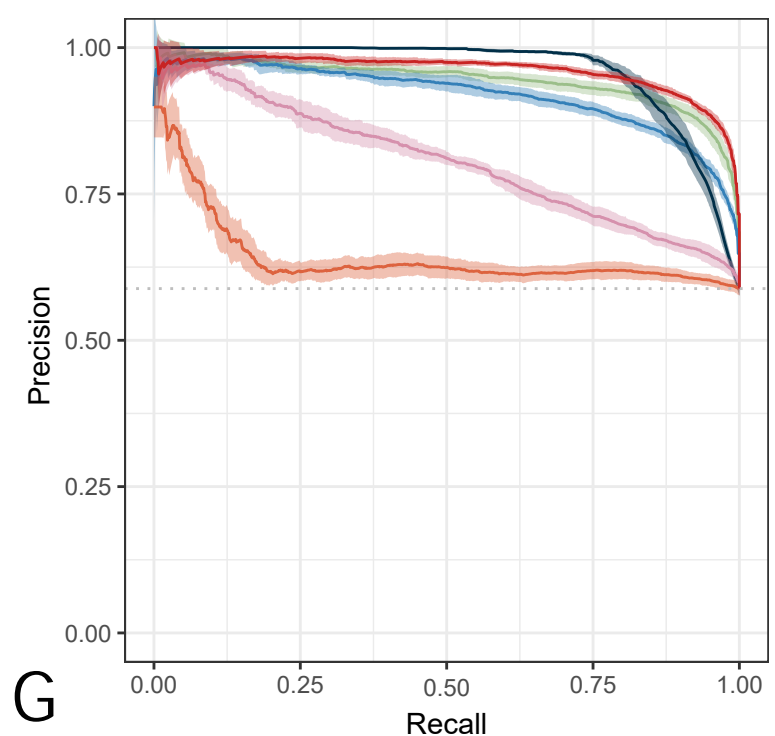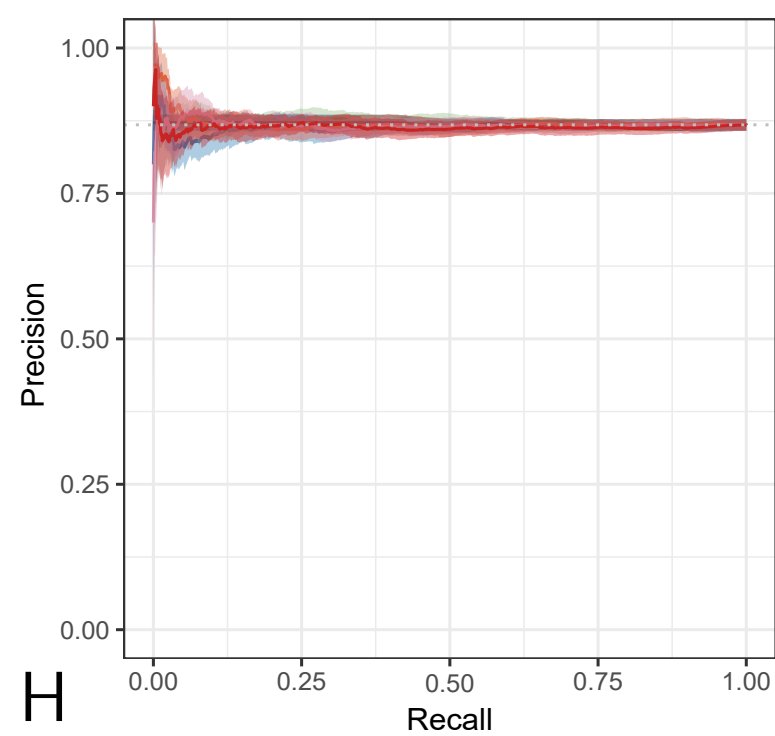

Supplement: Supplementary file 12 — Figure S12: Comparison of model performance before and after SMOTE processing. (A) ROC curves for models incorporating demographic characteristics and dietary micronutrients after SMOTE processing; (B) ROC curves for models incorporating demographic characteristics and dietary micronutrients without SMOTE processing; (C) PR curves for models incorporating demographic characteristics and dietary micronutrients after SMOTE processing; (D) PR curves for models incorporating demographic characteristics and dietary micronutrients without SMOTE processing; (E) ROC curves for models based on dietary micronutrients alone after SMOTE processing; (F) ROC curves for models based on dietary micronutrients alone without SMOTE processing; (G) PR curves for models based on dietary micronutrients alone after SMOTE processing; (H) PR curves for models based on dietary micronutrients alone without SMOTE processing. [file FSN3-14-e72019-s017.pdf]
