# Supplementary material for: Interpretable Machine Learning for Predicting Metabolic Syndrome–Kidney Stone Disease Comorbidity: The Role of Dietary Micronutrients
Source: Food Sci Nutr. 2026 Jun 10;14(6):e72019. doi: 10.1002/fsn3.72019 (PMC13253607; doi:10.1002/fsn3.72019)
Supplement: Supplementary file 7 — Figure S7: LIME‐based local interpretation for the Random Forest model incorporating demographic and dietary variables. (A) Local prediction probability for the illustrative case. (B) Local feature contribution plot. [file FSN3-14-e72019-s014.pdf]

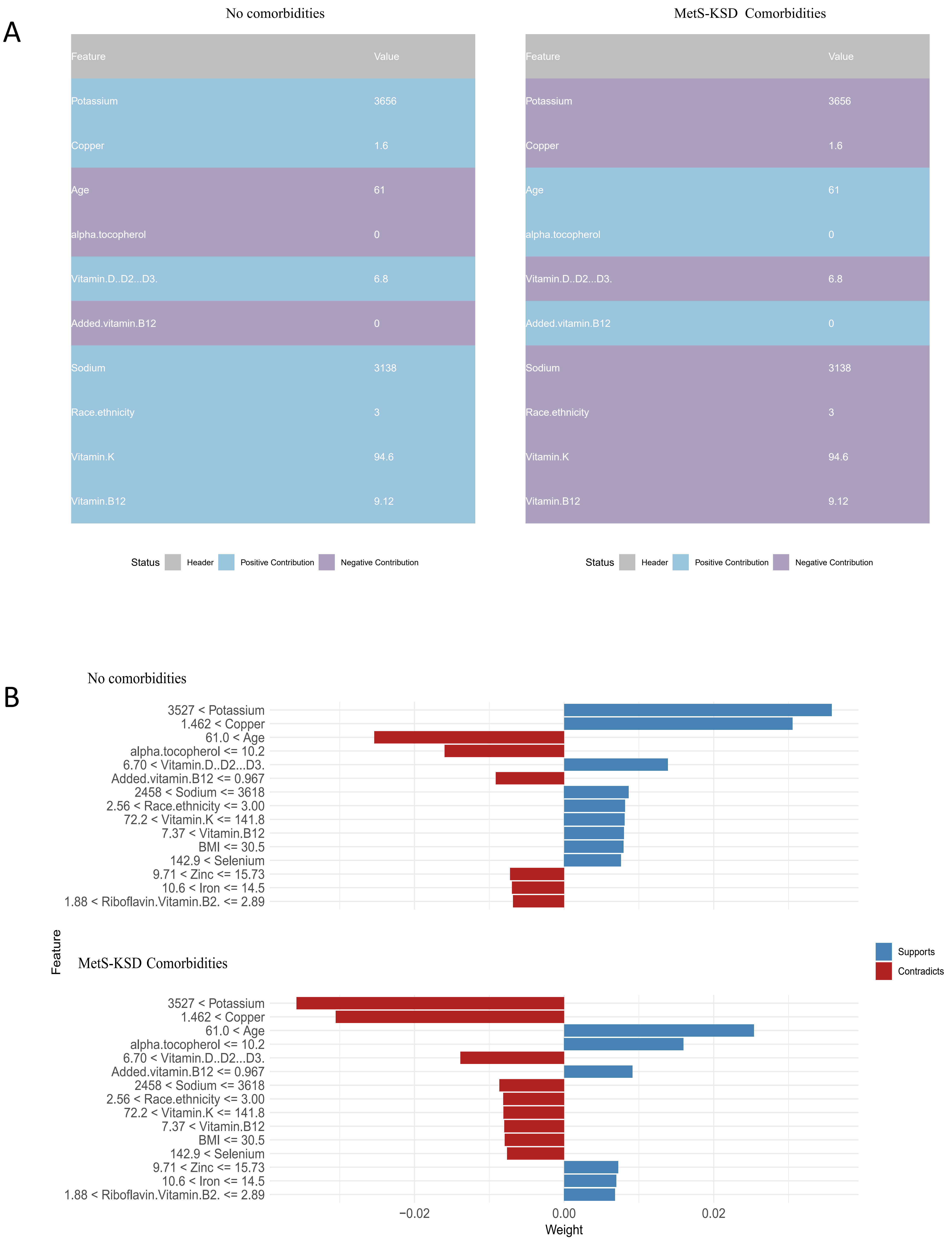
A
No comorbidities
Feature
Value
Potassium
3656
Copper
1.6
Age
61
alpha.tocopherol
0
Vitamin.D..D2...D3.
6.8
Added.vitamin.B12
0
Sodium
3138
Race.ethnicity
3
Vitamin.K
94.6
Vitamin.B12
9.12
Status
Header
Positive Contribution
Negative Contribution
MetS-KSD Comorbidities
Feature
Value
Potassium
3656
Copper
1.6
Age
61
alpha.tocopherol
0
Vitamin.D..D2...D3.
6.8
Added.vitamin.B12
0
Sodium
3138
Race.ethnicity
3
Vitamin.K
94.6
Vitamin.B12
9.12
Status
Header
Positive Contribution
Negative Contribution
B
No comorbidities
3527 < Potassium
1.462 < Copper
61.0 < Age
alpha.tocopherol <= 10.2
6.70 < Vitamin.D..D2...D3.
Added.vitamin.B12 <= 0.967
2458 < Sodium <= 3618
2.56 < Race.ethnicity <= 3.00
72.2 < Vitamin.K <= 141.8
7.37 < Vitamin.B12
BMI <= 30.5
142.9 < Selenium
9.71 < Zinc <= 15.73
10.6 < Iron <= 14.5
1.88 < Riboflavin.Vitamin.B2. <= 2.89
Feature
MetS-KSD Comorbidities
3527 < Potassium
1.462 < Copper
61.0 < Age
alpha.tocopherol <= 10.2
6.70 < Vitamin.D..D2...D3.
Added.vitamin.B12 <= 0.967
2458 < Sodium <= 3618
2.56 < Race.ethnicity <= 3.00
72.2 < Vitamin.K <= 141.8
7.37 < Vitamin.B12
BMI <= 30.5
142.9 < Selenium
9.71 < Zinc <= 15.73
10.6 < Iron <= 14.5
1.88 < Riboflavin.Vitamin.B2. <= 2.89
−0.02
0.00
0.02
Weight
Supports
Contradicts
