# Supplementary material for: Interpretable Machine Learning for Predicting Metabolic Syndrome–Kidney Stone Disease Comorbidity: The Role of Dietary Micronutrients
Source: Food Sci Nutr. 2026 Jun 10;14(6):e72019. doi: 10.1002/fsn3.72019 (PMC13253607; doi:10.1002/fsn3.72019)
Supplement: Supplementary file 13 — Table S1: Comparative performance of six SMOTE‐balanced machine‐learning models for predicting MetS–KSD comorbidity using demographic variables and dietary micronutrients. [file FSN3-14-e72019-s008.docx]

**Supplementary Table S1. Comparative performance of six SMOTE-balanced machine-learning models for predicting MetS–KSD comorbidity using demographic variables and dietary micronutrients.**

| **Model** | **Accuracy** | **F-beta** | **Area under the ROC curve** | **Sensitivity** | **Specificity** | **Area under the PR curve** |
| --- | --- | --- | --- | --- | --- | --- |
| Random Forest | 0.907 | 0.926 | 0.958 | 0.985 | 0.796 | 0.961 |
| Light GBM | 0.902 | 0.922 | 0.932 | 0.987 | 0.781 | 0.931 |
| KNN | 0.802 | 0.800 | 0.929 | 0.674 | 0.986 | 0.956 |
| Naive Bayes | 0.503 | 0.411 | 0.638 | 0.295 | 0.800 | 0.689 |
| SVM | 0.795 | 0.830 | 0.870 | 0.851 | 0.715 | 0.898 |
| XGBoost | 0.910 | 0.927 | 0.946 | 0.973 | 0.820 | 0.945 |
| *P* | <0.001^a^ | <0.001^a^ | <0.001^b^ | <0.001^a^ | <0.001^a^ | <0.001^a^ |
| ^a^ANOVA test; ^b^Kruskal-Wallis | | | | | | |
