# Supplementary material for: Interpretable Machine Learning for Predicting Metabolic Syndrome–Kidney Stone Disease Comorbidity: The Role of Dietary Micronutrients
Source: Food Sci Nutr. 2026 Jun 10;14(6):e72019. doi: 10.1002/fsn3.72019 (PMC13253607; doi:10.1002/fsn3.72019)
Supplement: Supplementary file 14 — Table S2: Comparative performance of six SMOTE‐balanced machine‐learning models for predicting MetS–KSD comorbidity using dietary micronutrients alone. [file FSN3-14-e72019-s012.docx]

**Supplementary Table S2. Comparative performance of six SMOTE-balanced machine-learning models for predicting MetS–KSD comorbidity using dietary micronutrients alone.**

| **Model** | **Accuracy** | **F-beta** | **Area under the ROC curve** | **Sensitivity** | **Specificity** | **Area under the PR curve** |
| --- | --- | --- | --- | --- | --- | --- |
| Random Forest | 0.881 | 0.904 | 0.938 | 0.949 | 0.786 | 0.944 |
| Light GBM | 0.838 | 0.868 | 0.905 | 0.905 | 0.742 | 0.919 |
| KNN | 0.807 | 0.804 | 0.932 | 0.677 | 0.993 | 0.961 |
| Naive Bayes | 0.473 | 0.376 | 0.556 | 0.270 | 0.762 | 0.641 |
| SVM | 0.678 | 0.748 | 0.743 | 0.813 | 0.488 | 0.805 |
| XGBoost | 0.896 | 0.911 | 0.956 | 0.913 | 0.871 | 0.960 |
| *P* | <0.001^a^ | <0.001^a^ | <0.001^b^ | <0.001^a^ | <0.001^a^ | <0.001^a^ |
| ^a^ANOVA test; ^b^Kruskal-Wallis | | | | | | |
