# Supplementary material for: Interpretable Machine Learning for Predicting Metabolic Syndrome–Kidney Stone Disease Comorbidity: The Role of Dietary Micronutrients
Source: Food Sci Nutr. 2026 Jun 10;14(6):e72019. doi: 10.1002/fsn3.72019 (PMC13253607; doi:10.1002/fsn3.72019)
Supplement: Supplementary file 15 — Table S3: Performance of reduced‐feature machine‐learning models constructed using the top 15 SHAP‐ranked variables under two modeling strategies. [file FSN3-14-e72019-s001.docx]

**Supplementary Table S3. Performance of reduced-feature machine-learning models constructed using the top 15 SHAP-ranked variables under two modeling strategies**

| **Model** | **Accuracy** | **F Beta** | **Area under the ROC curve** | **Sensitivity** | **Specificity** | **Area under the PR curve** |
| --- | --- | --- | --- | --- | --- | --- |
| Dietary micronutrients | |  |  |  |  |  |
| Random Forest | 0.831 | 0.865 | 0.900 | 0.918 | 0.707 | 0.914 |
| Light GBM | 0.810 | 0.846 | 0.882 | 0.885 | 0.704 | 0.904 |
| KNN | 0.775 | 0.774 | 0.885 | 0.659 | 0.939 | 0.922 |
| Naive Bayes | 0.475 | 0.374 | 0.561 | 0.267 | 0.772 | 0.644 |
| SVM | 0.666 | 0.740 | 0.717 | 0.810 | 0.459 | 0.780 |
| XGBoost | 0.859 | 0.880 | 0.929 | 0.885 | 0.822 | 0.943 |
| *P* | <0.001^a^ | <0.001^a^ | <0.001^b^ | <0.001^a^ | <0.001^a^ | <0.001^a^ |
| Demographic variables and dietary micronutrients | | | |  |  |  |
| Random Forest | 0.896 | 0.918 | 0.937 | 0.988 | 0.765 | 0.936 |
| Light GBM | 0.899 | 0.920 | 0.920 | 0.988 | 0.773 | 0.917 |
| KNN | 0.832 | 0.842 | 0.920 | 0.758 | 0.938 | 0.947 |
| Naive Bayes | 0.619 | 0.634 | 0.680 | 0.563 | 0.698 | 0.722 |
| SVM | 0.805 | 0.842 | 0.880 | 0.886 | 0.689 | 0.902 |
| XGBoost | 0.893 | 0.914 | 0.920 | 0.963 | 0.793 | 0.919 |
| *P* | <0.001^a^ | <0.001^a^ | <0.001^b^ | <0.001^a^ | <0.001^a^ | <0.001^a^ |
| ^a^ANOVA test; ^b^Kruskal-Wallis | | | | | | |
