# Supplementary material for: Interpretable Machine Learning for Predicting Metabolic Syndrome–Kidney Stone Disease Comorbidity: The Role of Dietary Micronutrients
Source: Food Sci Nutr. 2026 Jun 10;14(6):e72019. doi: 10.1002/fsn3.72019 (PMC13253607; doi:10.1002/fsn3.72019)
Supplement: Supplementary file 16 — Table S4: Performance of LASSO‐based reduced‐feature machine‐learning models under two modeling strategies. [file FSN3-14-e72019-s015.docx]

**Supplementary Table S4.** **Performance of LASSO-based reduced-feature machine- learning models under two modeling strategies**

| **Model** | **Accuracy** | **F Beta** | **Area under the ROC curve** | **Sensitivity** | **Specificity** | **Area under the PR curve** |
| --- | --- | --- | --- | --- | --- | --- |
| Dietary micronutrients | |  |  |  |  |  |
| Random Forest | 0.868 | 0.892 | 0.931 | 0.933 | 0.774 | 0.938 |
| Light GBM | 0.831 | 0.862 | 0.902 | 0.898 | 0.736 | 0.917 |
| KNN | 0.813 | 0.813 | 0.931 | 0.691 | 0.987 | 0.960 |
| Naive Bayes | 0.475 | 0.368 | 0.550 | 0.260 | 0.782 | 0.638 |
| SVM | 0.652 | 0.738 | 0.717 | 0.833 | 0.394 | 0.784 |
| XGBoost | 0.887 | 0.903 | 0.950 | 0.900 | 0.867 | 0.959 |
| *P* | <0.001^a^ | <0.001^a^ | <0.001^b^ | <0.001^a^ | <0.001^a^ | <0.001^a^ |
| Demographic variables and dietary micronutrients | | | |  |  |  |
| Random Forest | 0.894 | 0.915 | 0.946 | 0.978 | 0.774 | 0.948 |
| Light GBM | 0.900 | 0.920 | 0.930 | 0.984 | 0.780 | 0.931 |
| KNN | 0.807 | 0.808 | 0.915 | 0.694 | 0.968 | 0.947 |
| Naive Bayes | 0.510 | 0.435 | 0.637 | 0.321 | 0.780 | 0.689 |
| SVM | 0.787 | 0.824 | 0.855 | 0.850 | 0.698 | 0.885 |
| XGBoost | 0.901 | 0.919 | 0.939 | 0.964 | 0.811 | 0.939 |
| *P* | <0.001^a^ | <0.001^a^ | <0.001^b^ | <0.001^a^ | <0.001^a^ | <0.001^a^ |
| ^a^ANOVA test; ^b^Kruskal-Wallis | | | | | | |
