# Supplementary material for: Interpretable Machine Learning for Predicting Metabolic Syndrome–Kidney Stone Disease Comorbidity: The Role of Dietary Micronutrients
Source: Food Sci Nutr. 2026 Jun 10;14(6):e72019. doi: 10.1002/fsn3.72019 (PMC13253607; doi:10.1002/fsn3.72019)
Supplement: Supplementary file 17 — Table S5: Performance of machine‐learning models trained without SMOTE processing under two modeling strategies. [file FSN3-14-e72019-s003.docx]

**Supplementary Table S5. Performance of machine-learning models trained without SMOTE processing under two modeling strategies**

| **Model** | **Accuracy** | **F Beta** | **Area under the ROC curve** | **Sensitivity** | **Specificity** | **Area under the PR curve** |
| --- | --- | --- | --- | --- | --- | --- |
| Dietary micronutrients | |  |  |  |  |  |
| Random Forest | 0.868 | 0.929 | 0.561 | 1.000 | 0.000 | 0.894 |
| LightGBM | 0.866 | 0.928 | 0.565 | 0.998 | 0.004 | 0.897 |
| KNN | 0.838 | 0.911 | 0.505 | 0.955 | 0.069 | 0.869 |
| Naive Bayes | 0.735 | 0.840 | 0.522 | 0.815 | 0.206 | 0.876 |
| SVM | 0.868 | 0.929 | 0.526 | 1.000 | 0.000 | 0.881 |
| XGBoost | 0.860 | 0.924 | 0.548 | 0.988 | 0.015 | 0.891 |
| *P* | <0.001^a^ | <0.001^a^ | <0.001^b^ | <0.001^a^ | <0.001^a^ | <0.001^a^ |
| Demographic variables and dietary micronutrients | | | |  |  |  |
| Random Forest | 0.868 | 0.929 | 0.511 | 1.000 | 0.000 | 0.873 |
| LightGBM | 0.866 | 0.928 | 0.504 | 0.997 | 0.005 | 0.873 |
| KNN | 0.832 | 0.908 | 0.502 | 0.953 | 0.043 | 0.870 |
| Naive Bayes | 0.753 | 0.848 | 0.504 | 0.839 | 0.182 | 0.871 |
| SVM | 0.868 | 0.929 | 0.496 | 1.000 | 0.000 | 0.869 |
| XGBoost | 0.859 | 0.924 | 0.498 | 0.988 | 0.009 | 0.869 |
| *P* | <0.001^a^ | <0.001^a^ | <0.001^b^ | <0.001^a^ | <0.001^a^ | <0.001^a^ |
| ^a^ANOVA test; ^b^Kruskal-Wallis | | | | | | |
